# Two Material Properties from One Wavelength‐Orthogonal Photoresin Enabled by a Monochromatic Laser Integrated Stereolithographic Apparatus (Mono LISA)

**DOI:** 10.1002/adma.202419639

**Published:** 2025-02-17

**Authors:** Xingyu Wu, Katharina Ehrmann, Ching Thye Gan, Benjamin Leuschel, Fred Pashley‐Johnson, Christopher Barner‐Kowollik

**Affiliations:** ^1^ School of Chemistry and Physics Centre for Materials Science Queensland University of Technology (QUT) 2 George Street Brisbane Queensland 4000 Australia; ^2^ Faculty of Engineering Queensland University of Technology (QUT) 2 George Street Brisbane Queensland 4000 Australia; ^3^ Institut de Science des Matériaux de Mulhouse (IS2M) CNRS – UMR 7361 Université de Haute‐Alsace 15 rue Jean Starcky Mulhouse 68057 France; ^4^ Polymer Chemistry Research Group Centre of Macromolecular Chemistry (CMaC) Department of Organic and Macromolecular Chemistry Faculty of Sciences Ghent University Krijgslaan 281‐S4 Ghent 9000 Belgium; ^5^ Institute for Applied Synthetic Chemistry Technische Universität Wien Getreidemarkt 9/163 Vienna 1060 Austria; ^6^ Institute of Nanotechnology (INT) Karlsruhe Institute of Technology (KIT) Hermann‐von‐Helmholtz‐Platz 1 76344 Eggenstein‐Leopoldshafen Germany; ^7^ Institute of Functional Interfaces (IFG) Karlsruhe Institute of Technology (KIT) Hermann‐von‐Helmholtz‐Platz 1 76344 Eggenstein‐Leopoldshafen Germany

**Keywords:** multi‐color 3D printing, multi‐material 3D printing, orthogonal photochemistry, photoresins, wavelength‐orthogonal 3D printing

## Abstract

Multi‐material printing has experienced critical advances in recent years, yet material property differentiation capabilities remain limited both with regard to the accessible properties – typically hard versus soft – and the achievable magnitude of differentiation. To enhance multi‐material printing capabilities, precise photochemical control during 3D printing is essential. Wavelength‐differentiation is a particularly intriguing concept yet challenging to implement. Notably, dual‐wavelength printing to fabricate hard and soft sections within one object has emerged, where one curing process is insensitive to visible light, while UV irradiation inevitably activates the entire resin, limiting true spatio‐temporal control of the material properties. Until now, pathway‐independent wavelength‐orthogonal printing has not been realized, where each wavelength exclusively triggers only one of two possible reactions, independent of the order in which the wavelengths are applied. Herein, a multi‐wavelength printing technique is introduced employing a tunable laser to monochromatically deliver light to the printing platform loaded with a fully wavelength‐orthogonal resin. Guided by photochemical action plots, two distinct wavelengths – each highly selective toward a specific photocycloaddtion reaction – are utilized to generate distinct networks within the photoresin. Ultimately, together with the printing technique, this orthogonally addressable photoresin allows fabricating multi‐material objects with degradable and non‐degradable properties, in a single fabrication step.

## Graphical abstract

A single object with dual properties – degradable and non‐degradable – is fabricated in a single print simply by switching the printing colors. The advanced multi‐material printing is enabled by the combination of a fully wavelength‐orthogonal photoresin and a monochromatic tunable laser printer, paving the way for precise multi‐material differentiation within a single print.

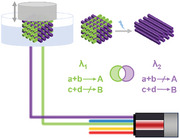

## 1 Introduction

Light‐driven additive manufacturing (AM) enables the fabrication of complex objects in a fast and efficient fashion. To date, a wide array of AM methods exploiting photochemical processes have been introduced, ranging from SLA (Stereolithography)^[^ ^1^ ^]^ to CLIP (Continuous Liquid Interface Production)^[^ ^2^ ^]^ for macroscopic objects to multi‐photon laser printing^[^ ^3^ ^]^ for microscopic and nano‐sized structures.^[^ ^4^ ^]^ Initially, these techniques were refined to rapidly print objects that feature one specific material property. Outstanding examples of rapid printing, intricate shapes, and practically applicable objects were realized and are able to be produced at industrial scales, making AM critical to the advancement of the fourth industrial revolution.^[^ ^5^ ^]^

However, modern‐day applications (e.g., medical prostheses, information storage devices) feature an intricate interplay of several material properties to acquire functionality, currently requiring the separate fabrication of device components and subsequent assembly into the multi‐property constructs.^[^ ^6^ ^]^ Thus, it would be highly beneficial to synthesize multi‐property objects via light‐driven 3D printing. Ideally, the operator of the 3D printer would not only select the geometry of the to be printed object during the design process, but at the same time assign a specific material property to each structural element.^[^ ^7^ ^]^

In light‐driven 3D printing, the ideal parameter to change is the color of light, i.e., the wavelength, or the photon flux, or a combination of both.^[^ ^8^ ^]^ The use of the color of light to selectively cure resists allows accessing disparate materials from a single resist simply by selecting a specific color of light. The key challenge in realizing such a scenario is to establish fully pathway independent wavelength orthogonality during the printing processes, i.e., a situation where one color of light exclusively triggers a specific reaction, whereas the other color of light independently triggers a different reaction, irrespective of each other's presence and the order in which the selective light triggers are applied.^[^ ^8^, ^9^ ^]^ The key component for selective material formation is that curing is “independent of the order”. Achieving such a scenario is challenging, considering that a photochemical process that is triggered with less energic (longer) wavelengths is very likely also triggered by more energetic (shorter) wavelengths. Thus, applying the shorter wavelength first in a photocuring process of a dual‐color responsive photoresin has a very high likelihood of triggering both photochemical processes within the resist – the one designed to be induced by less energetic light and the one designed to be triggered by higher energy light (semi‐orthogonal curing).^[^ ^10^ ^]^

Clearly, the above scenario is an undesirable situation, although it has been exploited in light driven 3D printing as we will explore below. The answer to pathway‐independent irradiation conditions and entirely selective curing of a mixture of two photoresins lies in understanding the wavelength resolved reactivity of each of the components.

Over the past decade, we have demonstrated that the absorption spectrum of a photoreactive chromophore is a very poor predictor for selecting the most effective wavelength for driving a reaction, i.e., determining at which wavelength the quantum yield of the reaction is highest. Indeed, our photochemical action plot methodology used to determine wavelength dependent photochemical reactivity (quantum yields) has demonstrated that the absorption spectrum often has limited resemblance with the wavelength dependent quantum yield.^[^ ^11^ ^]^ Inspection of the photochemical action plot of two reactive chromophores can determine near‐perfect orthogonality windows for fully pathway independent photocuring.^[^ ^12^ ^]^ Herein, we exploit such photochemical action plots and introduce the first pathway independent curable photoresin for light‐driven 3D printing that allows fabricating structures with different material properties from one resin, while the order in which each color of light is used is irrelevant.

Early attempts of multi‐property photochemically driven 3D printing utilized engineering solutions, where a mechanism transfers printed objects between vats in the middle of the process or where the material source is switched/altered mid‐process.^[^ ^6^, ^13^ ^]^ These strategies are highly laborious, making precise 3D control challenging. The choice of positions for different material properties within the synthesized object is limited, making frequent switching difficult and even impossible switching within one printing layer. These limitations inhibit freedom of object design and thus functionality. The arguably most advanced multi‐property synthesis strategy utilizes inkjet printing with several nozzles for multi‐property parts.^[^ ^13e,g,h^ ^]^ This achievement features, however, limited material property differentiation, relying on additives in classical (meth)acrylic photosensitive resins that are limited in concentration due to ink viscosity. In addition, the diffusion of inks into already printed sections further limits property differentiation.^[^ ^13e^ ^]^

**Scheme 1:**
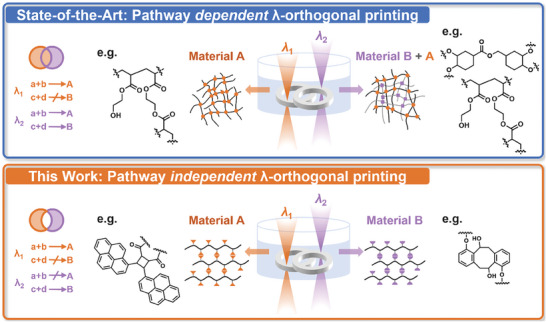
Previous dual‐wavelength multi‐material printing based on pathway‐dependent wavelength orthogonal network formation of material A with λ_1_ (radical polymerization) and material A + B with λ_2_ (radical and cationic polymerization) (top), whereas the current work pioneers pathway‐independent, fully wavelength orthogonal printing of material A with λ_1_ and material B with λ_2_ using step‐growth polymerization reactions (bottom).

As alluded to above, the use of two colors of light has been explored to deliver hard and soft sections in one object via two so‐called semi‐orthogonal or pathway‐dependent wavelength orthogonal reactions. In all cases, one curing process is insensitive to visible light irradiation but initiation of the other component in the resin during harsher UV‐irradiation cannot be avoided, resulting in the formation of an interpenetrating network during UV‐irradiation.^[^ ^10^, ^14^ ^]^ This semi‐orthogonality excludes the possibility to print exclusively only one out of two materials at each wavelength and thus limits the achievable multi‐material properties and functionalities, restricting true spatiotemporal control within a single construct. Only a few semi‐orthogonal examples exist, most of them based on the same polymer classes (**Scheme** **1** **top**):^[^ ^10^, ^14^ ^]^ Radical photopolymerization of (meth)acrylates cured with visible light through commercially available photoinitiators, which are active in both UV‐ and vis‐range, delivers soft networks. In combination with cationically UV‐cured epoxy networks utilizing photoacid generators, which are only active in the UV‐range, hard interpenetrating networks (IPNs) are produced due to simultaneous reaction of both photoinitiators upon UV light irradiation. In one example, selective concealing of photoinitiators by photoswitches (“solution masks”) has also been utilized in a process called solution mask liquid lithography (SMaLL), effectively leading to the same outcome.^[^ ^10^, ^14^ ^]^ Two other examples are based on thiol‐acrylate step‐growth polymerization and radical polymerization of acrylates. In one example, exposure to visible light (405 nm) induces rapid curing through a combination of radical polymerization of acrylates and thiol‐acrylate polymerization, and irradiation with UV light (365 nm) additionally triggers the [2 + 2] photocycloaddition of coumarin chromophores.^[^ ^14c^ ^]^ In another example, exposure of the resin to UV light (365 nm) results in the formation of a rigid network via a combination of radical chain‐growth and thiol‐acrylate step‐growth mechanisms, whereas exposure to visible light (405 nm) forms a soft network predominantly through an anionic thiol‐acrylate step‐growth mechanism.^[^ ^14d^ ^]^ While the number of reports on hard/soft multi‐property printing has recently been increasing,^[^ ^8^, ^10^, ^15^ ^]^ differentiation of properties beyond these are still missing.

To the best of our knowledge, only one example exists for pathway independent wavelength orthogonal polymer network synthesis from one photoresin – albeit not in the realm of 3D printing – which is based on step‐growth polymerization through uncatalyzed photocycloadditions.^[^ ^12g^ ^]^ Two wavelength‐orthogonal chromophores are used as prepolymer‐pendant crosslinkable functional groups. Upon irradiation, uncatalyzed photocycloadditions led to selective crosslinking, forming two polymer networks with two colors of light. Maintaining orthogonality within the above system relies on the cycloreversion of the styrylpyrene‐chromophore cycloadduct in the UV‐range in combination with different kinetics of the reaction. Notably, the approach has only been demonstrated in 2D‐photomask synthesis and has never been translated to light‐driven 3D printing, due to two main limitations to accessing full orthogonality in vat photopolymerization: *i*) Resins are required to exhibit fast crosslinking and their dilution with solvents must remain very limited for 3D printing. The use of chromophores makes it difficult to maintain orthogonality under non‐dilute, neat photopolymerization conditions. Under bulk synthesis conditions, the high chromophore concentrations cause cross‐sensitization between two chromophores, which should react to two different wavelength regimes, as we discovered earlier.^[^ ^16^ ^]^ *ii*) The lack of 3D printers with high freedom of wavelength‐choice restricts precise wavelength‐window choices available to 3D printing until now.

Inspired by the aforementioned foundational studies and recent advances in wavelength‐orthogonal reaction mechanisms,^[^ ^10^, ^12^ ^]^ we herein close these critical gaps in light‐driven printing by pioneering the first fully wavelength‐selective dual‐resist system based on *pathway‐independent orthogonality*, capable of independently curing specific materials by modulating the wavelength. In our system, one wavelength can only trigger one of two possible reactions, and the second wavelength exclusively triggers the second reaction (Scheme 1 **bottom**). The energetically higher wavelength does not induce crosslinking of the reactive moieties designed for the longer wavelength, which is a substantial advancement over the current state of the art. This printing is enabled by our own developed advanced 3D printer – the **Mono**chromatic Tuneable **L**aser **I**ntegrated **S**tereolithographic **A**pparatus (Mono LISA) – which addresses the key technological challenge. As a result, degradable and non‐degradable materials can be printed macroscopically simply by changing the color of light during the printing process.

## 2 Results and Discussion

### 2.1 Mono LISA

**Figure 1:**
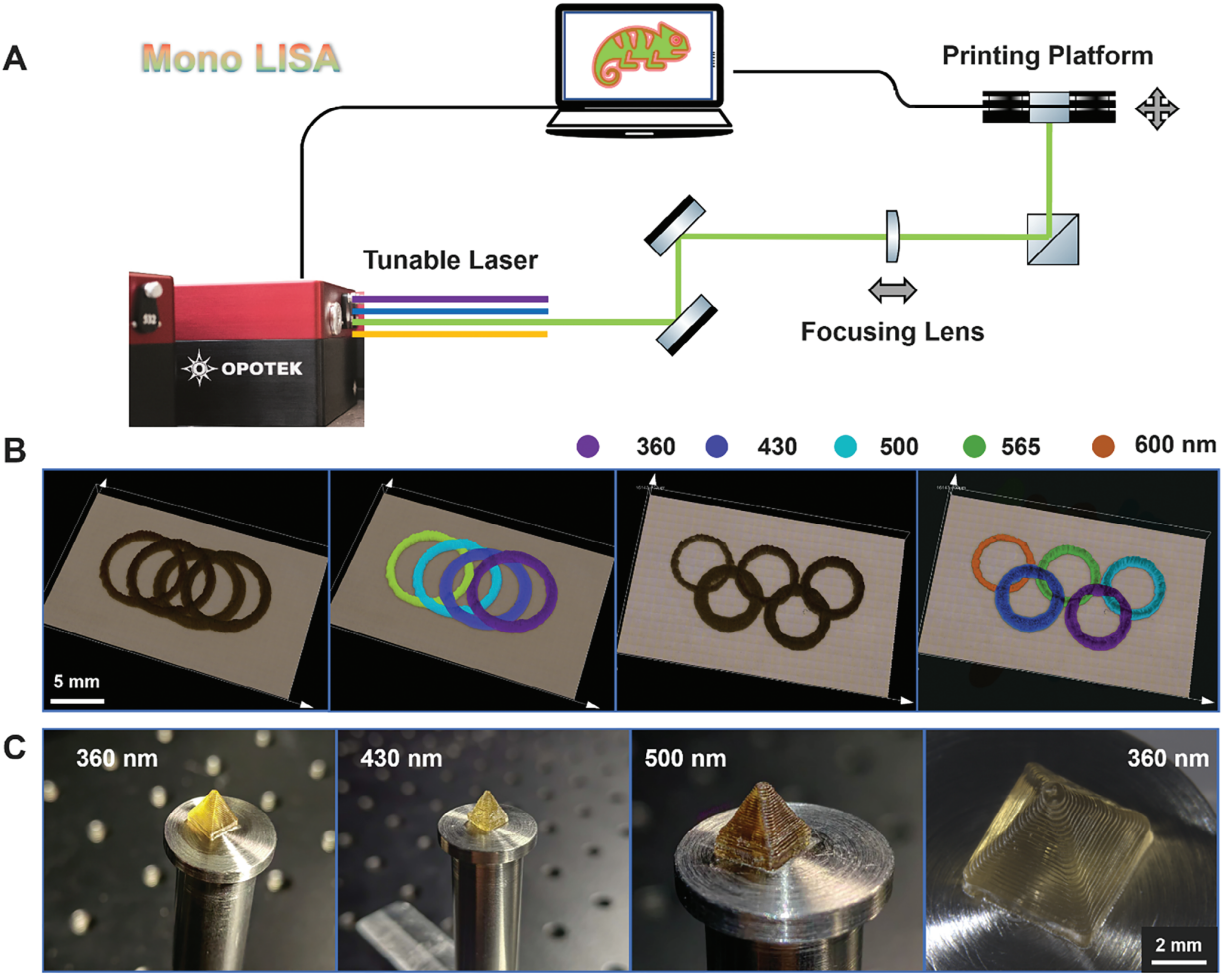
A) Schematic of the Mono LISA printer, along with an illustration of the output beam pathway from the tunable laser to the printing platform. B) Interlocked rings printed at multiple wavelengths in a single print using an acrylate‐based resin with ZnTPP as a photosensitizer (left) and their false‐color images reflecting the printing wavelengths (right). C) Photographs of pyramids printed at 360, 430, and 500 nm, accompanied by a microscopic image of the pyramid printed at 360 nm showing detailed features.

To achieve pathway independent, fully orthogonal 3D printing with two colors of light, two technological aspects must be ensured: Access to a broad range of wavelengths and intensities, and software‐facilitated switching of these parameters. While some dual‐color digital light processors have become available, these printers are restricted to switching between two LED arrays generally utilizing UV and blue light.^[^ ^10a^ ^]^ Their limited range of accessible wavelengths and spectral distribution which blurs the differentiation of the reactivities of two chromophores, significantly reduces their versatility. Therefore, we developed an advanced 3D printing system, the Mono LISA, by integrating our monochromatic tunable laser system (wavelength range of 210–2400 nm) with a custom‐designed 3D printing platform. As shown in **Figure** **1**A, the laser beam reflects off UV‐enhanced aluminum mirrors, passes through a focusing lens, and is ultimately directed to a 3‐axis stage (X, Y, and Z) using a UV silica right‐angle prism. Upon importing G‐code files – containing the coordinates and travel speed – into our customized software (File S1, Supporting Information), the software processes these files to precisely control both the printing trajectory and speed, enabled by the movement of the 3‐axis stage. The specifications of the optics and other components of our setup as well as the photographs and design of the 3D printing platform are provided in Section 3.1, Scheme S1, and Figure S1 (Supporting Information). The G‐codes for printing and their corresponding trajectories can be found in Files S2–S4 and Figure S2 (Supporting Information), respectively.

To demonstrate the effectiveness of our wavelength‐resolved printing approach, a radical‐based photoresin with a broad range of activatable wavelengths was formulated by combining pentaerythritol triacrylate (PETA), a trifunctional acrylate, with zinc tetraphenyl porphyrin (ZnTPP) as the sensitizer.^[^ ^17^ ^]^ We first determined the feature sizes of lines printed at different wavelengths and laser powers (Figure S3, Supporting Information). A positive correlation of line width and height with laser power, and a negative correlation of line width with wavelength were revealed (Figures S4 and S5, Supporting Information), suggesting that the photoreactivity of ZnTPP varies with wavelength, an effect that can be further investigated using our developed photochemical action plot method^[^ ^11^ ^]^ and the Mono LISA system. Based on these feature sizes, interlocking rings were successfully printed using a range of wavelengths from 360 to 600 nm in a single print (Figure 1B). The spatial positioning of the rings perfectly matched the encoding in the G‐code files (Files S2 and S3, Figure S2, Supporting Information). More intriguingly, three well‐defined, high‐resolution pyramids (Figure 1C) were printed at wavelengths of 360, 430, and 500 nm, further demonstrating the remarkable capabilities of our printer. It must be stressed here that the demonstrated wavelengths are not exhausting the printer's capabilities, which can provide any wavelength between 210 and 2400 nm. Based on our 3D printing results, any wavelength between 360 and 500 nm can be utilized for 3D printing, with printing speed and quality only limited by the maximum available irradiation intensity at each of the laser's excitation wavelengths. It is also important to note that – in addition to laser wavelength and power – our Mono LISA enables the adjustment of printing speeds for each pathway facilitated by the software. Herein, we selected a printing speed of 0.1 mm s^−1^ considering our current hardware setup, which includes a laser with a repetition rate of 20 Hz and a mechanical stage, aligned with our intention to minimize power requirements. The relatively slow printing speed could be enhanced by replacing the mechanical stage with a mirror‐based system, such as a galvo scanner, and employing a higher laser repetition rate. The G‐code for the pyramid and its trajectory are provided in File S4 and Figure S2 (Supporting Information) respectively. All ZnTPP‐based printing process and parameters can be found in the Supporting Information (section 3.2.). Regarding the power measurements – as noted in the Supporting Information (Section 3.1.) – we measured the power directly at the beam output (Scheme S1, Supporting Information). However, we are also aware of the importance of the intensity at the printing site as well as the intensity distribution which could be correlated with the lateral feature sizes. Therefore, the beam sizes and intensities at the printing site correlated with the output power as well as the spatial mappings of the laser spot at the printing site have been provided in Table S1 and Figure S6 (Supporting Information), respectively.

### 2.2 Pathway Independent Wavelength‐Orthogonal Printing

Taking advantage of the versatility of our printer, in the following section, we describe the working principles of our developed fully wavelength‐orthogonal (pathway‐independent orthogonal) photochemical reactions and their printability, before progressing to their implementation in a dual‐resist system and ultimately to the ability of our resist to be cured independently into disparate, spatially resolved soft matter materials by using two different wavelengths.

#### 2.2.1 Single‐Resist‐Based Printing

**Figure 2:**
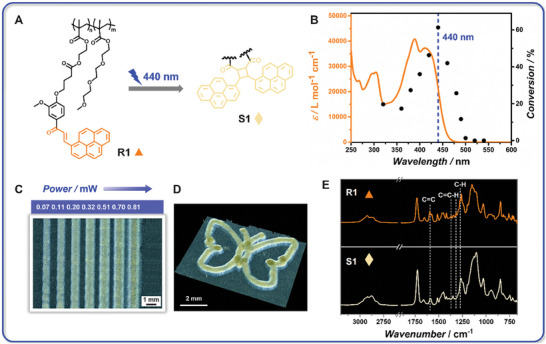
A) The Py‐Chal part in **R1** undergoes [2 + 2] cycloaddition under long‐wavelength irradiation (e.g., 440 nm) for the formation of network **S1**. B) Photochemical action plot of Py‐Chal (black dots) plotted against the UV–vis absorption spectrum of Py‐Chal (orange line).^[^ ^19^ ^]^ The blue dash line indicates the selected wavelength for printing. C) Microscopic image of lines printed from **R1**‐based photoresist with increasing laser power *P* at 440 nm (printing speed 0.03 mm s^−1^). D) Microscopic image of a butterfly structure printed at 440 nm with *P* = 0.11 mW (printing speed 0.03 mm s^−1^). E) FTIR spectra of **R1** and **S1** (printed structure) with dashed lines highlighting the changes of the characteristic peaks of **R1** after irradiation.

We base our dual‐resist system on two wavelength‐orthogonal chemistries: *i*) Poly‐(Py‐Chal)_n_‐*co*‐TEGMA_m_ (n:m = 3:7, *M*_n_ = 3900, *Đ* = 1.60), referred to as **R1**, which serves as the long‐wavelength‐active species; and *ii*) Poly‐(*o*MBA)_n_‐*co*‐MMA_m_ (n:m = 7:27, *M*_n_ = 5360, *Đ* = 1.65), referred to as **R2**, which constitutes the short‐wavelength‐active species. The synthesis and characterization of the chemical structures of Py‐Chal, *o*MBA, and their corresponding monomers and prepolymers have been provided in the Supporting Information (Schemes S2–S5 and Figures S7–S11, Supporting Information). In **R1** (**Figure** **2**A), pyrene‐chalcone (Py‐Chal) can undergo a [2 + 2] photocycloaddition, making it a promising candidate as a crosslinking unit for 3D printing.^[^ ^18^ ^]^ Interestingly, the resulting dimer can be reverted to the monomer, offering potential for post‐printing photodegradation.^[^ ^19^ ^]^ In addition, poly‐TEGMA exhibits a low glass transition temperature (*T*_g_) and hydrogel‐like behavior, which facilitates the diffusion of solvent into the printed structure and further enhances the degradation process.^[^ ^20^ ^]^ The photodegradation aspect will be addressed later.

In light‐based printing, the penetration depth of light and photochemical reactivity need to be considered, as these are highly dependent on the absorptivity of the resist and the wavelength‐dependent quantum yield of the photoactive species. Our group has discovered that the absorption spectrum of a chromophore provides inadequate guidance for its chemical reactivity – in other words – that there is a fundamental mismatch between photochemical reactivity and absorptivity, using so‐called photochemical action plots for bond forming and cleaving reactions.^[^ ^11^ ^]^ Photochemical action plots depict the yield of a photochemical reaction as a function of the incident wavelength at a constant photon count (the setup is illustrated in Figure S12, Supporting Information). Inspection of the UV–vis absorption spectrum and the action plot for the photocycloaddition of Py‐Chal confirm that photochemical reactivity and absorbance are not congruent, as our group has observed across a broad range of photochemical reactions.^[^ ^11^ ^]^ As shown in Figure 2B, the highest reactivity occurs at 440 nm, which is red‐shifted by close to 15 nm from the absorption maximum (λ_max_ = 425 nm).^[^ ^19^ ^]^ This red‐shifted reactivity is advantageous in light‐based printing, as the lower molar extinction coefficient at this wavelength promotes deeper light penetration while maintaining high reactivity.^[^ ^21^ ^]^ Critically, Py‐Chal photodimerization is efficiently suppressed at 320–360 nm, allowing for orthogonal printing with UV‐active resins, specifically **R2**, which will be discussed subsequently. To implement the presented photochemistry in a macromolecule‐based dual‐resist system, it is essential that the long‐wavelength‐active resin exhibits low reactivity or poor printability in the UV range, and vice versa. Furthermore, the dimer exhibits strong absorption at 320 to 360 nm, promoting the cycloreversion of the dimer, thereby facilitating degradation.^[^ ^19^, ^22^ ^]^

For long‐wavelength‐based printing, a Py‐Chal‐based photoresist (**R1**: propylene carbonate = 60:40 wt.%) has been established, with 440 nm selected as the optimal wavelength, leveraging the high reactivity and potential deep penetration depth of Py‐Chal. As **R1** is highly viscous, the careful selection of a solvent to achieve the desired viscosity is an important issue for printing. Propylene carbonate, with its high solubility and low volatility, can adjust the viscosity of printing formulations, ensuring optimal flow and deposition. We screened a range of laser powers from 0.07 to 0.81 mW for line formation at a printing speed of 0.03 mm s^−1^. Printing with **R1** is readily achievable, as indicated by its broad fabrication window (Figure 2C). As shown in Figure 2C, the line width increases from 669.6 to 1122.5 µm as the laser power increases from 0.07 to 0.81 mW. We anticipate that higher laser power drives more [2 + 2] reactions, resulting in wider lines after the development step with acetone to remove the soluble constituents.^[^ ^18^ ^]^ A more intricate and well‐defined curvy structure (e.g., butterfly) was printed, demonstrating the excellent printability of **R1** and further highlighting the capability of the Mono LISA (Figure 2D). The G‐code for the butterfly and its trajectory are provided in File S5 and Figure S13 (Supporting Information). In order to evidence the occurrence of the photocycloaddition reaction, FTIR spectra of **R1** and the fabricated structure from a single Py‐Chal‐based resist (**S1**) were recorded (Figure 2E). The absorption peaks in **R1** at 1597 cm^−1^ (C═C), 1372 and 1316 cm^−1^ (C═C─H), and 1275 cm^−1^ (C─H), corresponding to the chalcone groups, show notable changes in the fabricated structure **S1**.^[^ ^23^ ^]^ Specifically, the C═C peak decreases, the C═C─H peaks decrease or even disappear, and the C─H peak increases, strongly suggesting the [2 + 2] dimerization of Py‐Chal. While it would be interesting to analyze network formation of our photoresists with techniques such as photorheology or photo‐DSC, the currently available light sources for such instruments do not allow replication of the exact irradiation conditions required for our printing. Therefore, we have opted for ex‐situ FTIR spectroscopy after irradiation of the resins. Additionally, the mechanical behavior of **S1** was evaluated through nanoindentation experiments. The measured modulus and hardness were 23.92 ± 1.34 MPa and 0.27 ± 0.02 MPa (N = 16), respectively (Figure S14, Supporting Information). These relatively low values are likely related to the low glass transition temperature (*T*_g_) of poly‐TEGMA incorporated into the Py‐Chal‐based copolymer.

**Figure 3:**
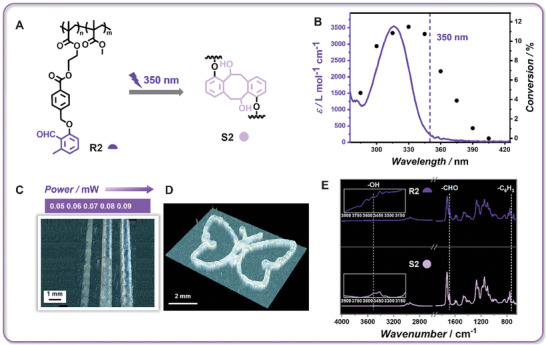
A) The *o*MBA part in **R2** undergoes phototautomerization to a diene under short‐wavelength irradiation (e.g., 350 nm), which readily undergoes a [4 + 4] cycloaddition enabling network formation for **S2**. B) Photochemical action plot for the cycloaddition of *o*MBA (black dots) plotted versus the UV–vis absorption spectrum of *o*MBA (purple line).^[^ ^25^ ^]^ The purple dash line indicates the selected wavelength for printing. C) Microscopic image of lines printed from **R2**‐based photoresist with increasing laser power *P* at 350 nm (printing speed 0.03 mm s^−1^). D) Microscopic image of a butterfly structure printed at 350 nm with *P* = 0.07 mW (printing speed 0.03 mm s^−1^). E) FTIR spectra of **R2** and **S2** (printed structure) with dashed lines highlighting the changes of the characteristic peaks of **R2** after irradiation.

We now turn to the short‐wavelength‐active **R2** (**Figure** **3**A). The diene generated from an *o*‐methyl benzaldehyde (*o*MBA) species (present in **R2**), capable of efficient dimerization, is highly effective for complex polymer synthesis and photocuring applications.^[^ ^12^, ^24^ ^]^ In contrast to TEGMA in **R1**, the incorporation of MMA into **R2** enhances the rigidity of the network formed by the dimerization of *o*MBA, providing a distinct contrast to the degradable Py‐Chal network. Inspection of the photochemical action plot for the dimerization of *o*MBA reveals a mismatch between UV‐v is absorption and dimerization efficiency (Figure 3B).^[^ ^25^ ^]^ The high reactivity observed between 320 and 350 nm contrasts with Py‐Chal, whose reactivity in this range is significantly suppressed. Furthermore, the reactivity of *o*MBA above 415 nm is negligible, whereas Py‐Chal exhibits high reactivity, enabling the orthogonal dimerization of Py‐Chal and *o*MBA through selective wavelength control.

An **R2**‐based photoresist (**R2**: propylene carbonate: acetophenone = 50:25:25 wt.%) was established with propylene carbonate introduced as a solvent to maintain consistency with the **R1**‐based photoresist, and acetophenone to enhance the solubility of solid **R2**. 350 nm was selected for printing. At a printing speed of 0.03 mm s^−1^ – consistent with that used for **R1** – a well‐defined line with a width of 620 µm forms at 0.07 mW (Figure 3C). Below this power, no structures remain on the glass substrate after development, while above it, hollow structures appear, indicating proximity to the fabrication and damage thresholds. To further broaden the fabrication window, the concentration of **R2** was increased to 70%. However, the fabrication window remained unchanged and defects appeared in the structure (Figure S16, Supporting Information) due to the high viscosity of **R2**, which hindered uniform deposition. As a result, we maintained the optimal concentration at 50 wt.%. Despite the narrower fabrication window compared to **R1**‐based printing, intricate structures (e.g., a butterfly) can still be reliably produced (Figure 3D). During the printing process, the reactive diene species (*o*‐quinodimethane) is formed, followed by the dimerization, resulting in the consumption of aldehyde functional entities and the generation of hydroxyl groups.^[^ ^24a^ ^]^ This process is evidenced by the FTIR spectra of **R2** and the fabricated structure from a single *o*MBA‐based resist (**S2**) (Figure 3E). Absorption peaks corresponding to the aldehyde functional groups at 1687 cm^−1^ and the adjacent benzene group at 732 cm^−1^, clearly observed in **R2**, show a significant decrease in the spectrum of **S2**. Furthermore, a broad absorption peak ≈3513 cm^−1^ appears in the spectrum of **S2**, which can be assigned to the formed hydroxyl group.^[^ ^24a^ ^]^ These results clearly demonstrate that the dimerization of *o*MBA is responsible for successful **R2**‐based printing. The mechanical behavior of **S2** was evaluated as well, revealing a modulus and hardness of 4.45 ± 0.18 GPa and 94.17 ± 8.41 MPa (N = 16), respectively (Figure S14, Supporting Information). These values are 186 times and 348 times higher than those of **S1**. The substantial differences in mechanical properties can be attributed to the molecular design, where poly‐TEGMA was incorporated into **S1** and poly(MMA) into **S2**. Additionally, the substantial negative forces observed during the withdrawal of the indenter from the surface in indentation curve profiles (Figure S15, Supporting Information) reveal that **S1** (the Py‐Chal‐based printed structure) exhibits significantly stronger adhesive behavior compared to **S2**, further highlighting the versatility of our chemical platform in printing objects with diverse properties.

It is worth noting that both polymers used in **R1** and **R2** have low molecular weights (i.e., short polymer chains), facilitating the diffusion of non‐crosslinked or poorly crosslinked polymers from solid structures, which is particularly important in multi‐material printing, where precise control over material properties is essential.^[^ ^8a^ ^]^ Notably, both **R1**‐ and **R2**‐based photoresists are photoinitiator‐free, meaning they do not rely on traditional photoinitiators to initiate polymerization or crosslinking reactions. Instead, they employ step‐growth mechanisms, offering several advantages, including simplified formulation and improved homogeneity of the polymer network within the structure.^[^ ^26^ ^]^ In addition, by pre‐fabricating polymer chains that are subsequently photochemically crosslinked, the final properties of the object can be finely tuned through the incorporation of different monomers into the polymer chains.^[^ ^26^ ^]^

#### 2.2.2 Dual‐Resist‐Based Printing

**Figure 4:**
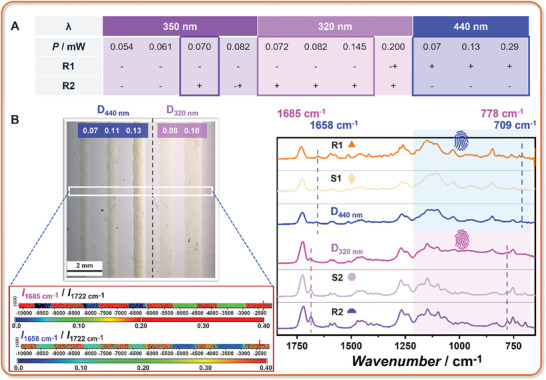
A) Printability of **R1**‐ and **R2**‐based photoresists across specific power ranges at 350 nm, 320 nm, and 440 nm (+, – and ± stand for print well, cannot print, and can be printed but not well, respectively; the frames suggest the selectivity windows at these wavelengths. Printing speed 0.03 mm s^−1^). B) Microscopic image of lines printed from a dual‐resist system with different laser powers at 440 and 320 nm (printing speed 0.03 mm s^−1^), along with the FTIR spectra of **D**_440_ _nm_ and **D**_320_ _nm_ compared to those of **R1**, **S1**, **R2**, and **S2**, demonstrating the two distinct materials printed at two different wavelengths from a single print. Peaks at 1658 and 709 cm^−1^ (blue dashed lines) are present only in Py‐Chal‐based prepolymer and structures. Peaks at 1685 and 778 cm^−1^ (pink dashed lines) are shown only in *o*MBA‐based prepolymer and structures. The fingerprint region in **D**_440_ _nm_ shows similarity to that in **R1** and **S1** (blue background), while the region in **D**_320_ _nm_ is closer to that of **R2** and **S2** (pink background). Chemical mapping with FTIR visualizes the distinct materials printed at two different wavelengths (left corner). One area across the five lines (marked with a white box) was scanned from left to right. The ratios *I*_1685 cm−1_/*I*_1722 cm−1_ and *I*_1658 cm−1_/*I*_1722 cm−1_ were used for mapping. The values are presented with color as indicated by the color scale bar. For *I*_1685 cm−1_/*I*_1722 cm−1_, the first three lines appear blue, while the other two are green. In contrast, for *I*_1658 cm−1_/*I*_1722 cm−1_, the colors are inverted, highlighting the distinct materials between the first three lines and the other two. The reddish color represents the substrate background.

To implement the two presented photoresists into a dual‐resist system, it is key that the reactivity of one species is not affected by the presence of the other photoreactive species. Analysis of the action plots of Py‐Chal and *o*MBA – along with the printability of **R1** and **R2** – suggests that 440 nm could serve as the long wavelength, while a wavelength between 320 and 360 nm could effectively be used as the short wavelength to achieve pathway independent wavelength‐orthogonal printing. Prior to exploring the dual‐resist system, the constitution of the **R1‐**based photoresist was optimized to further suppress its low reactivity at short wavelength, such as at 350 nm (the wavelength previously used for *o*MBA printing), as poor‐quality printing or no printing at all is expected for **R1** at these wavelengths. As shown in Figure S17 (Supporting Information), when the concentration of **R1** in the **R1**‐based photoresist is reduced from 60 to 30 wt.%, lines can no longer be printed at 0.07 mW (350 nm), which is the power required for fabricating high‐quality **R2**‐based structures. **Figure** **4**A summarizes the printability of **R1**‐ and **R2**‐based photoresists across specific power ranges at different wavelengths (related images are presented in Figure S18, Supporting Information). We define the selectivity window as the power range in which the reactivity of one resist is strongly enhanced, while that of the other is sufficiently suppressed. The wavelength orthogonality within the selectivity window enables fully wavelength‐orthogonal printing, provided that no cross‐sensitization or cross‐addition occurs. To further expand the selectivity window at short wavelengths, the printability of **R2** at 320 nm was investigated, resulting in a much wider selective window (0.072–0.145 mW) compared to the narrow window at close to 0.07 mW at 350 nm. The selectivity window at the long wavelength (440 nm), where lines cannot be printed with **R2**, while **R1** presents excellent printability, is broad. We emphasize that the high tunability of the Mono LISA critically facilitates the selection of the printing wavelength and power. Moreover, the poor printability of **R1** at 320 nm further indicates that the reverse [2 + 2] cycloaddition is favored, offering a unique opportunity for the photodegradation of Py‐Chal‐based structures under UV light. The beam sizes at the printing site at 320, 350, and 440 nm and the intensities at the printing site correlated with the output power as well as the spatial mappings of the laser spot at the printing site have been provided in Table S2 and Figure S19 (Supporting Information), respectively.

Encouraged by the results of the selectivity windows, we proceeded to dual‐resist‐based printing. A dual‐resist formulation (**R1**: **R2**: propylene carbonate: acetophenone = 25: 38: 18.5: 18.5 wt.%) was prepared; the excellent miscibility of **R1** and **R2** provides a solid foundation for subsequent printing. Based on the selectivity window, well‐defined lines were obtained from a single print using 440 nm (at 0.07–0.13 mW) for the first three lines and 320 nm (at 0.08–0.10 mW) for the last two lines (Figure 4B). We expect the use of these two distinct wavelengths, each highly selective toward a specific photocycloaddition reaction, to form respective networks within the photoresin.

To assess the wavelength‐orthogonal addressability of both photoreactive species, mixtures consisting of *o*MBA and Py‐Chal chromophores (2.6:1 mol%, consistent with the dual‐resist formulation) were irradiated at both wavelengths, 320 and 440 nm. Subsequent investigation using liquid chromatography mass spectrometry (LC‐MS, Figure S20, Supporting Information) revealed that Py‐Chal dimerization was exclusively initiated at 440 nm (with dimers eluting at 12.0 and 12.25 min),^[^ ^22^ ^]^ while at 320 nm, *o*MBA dimers were detected (eluting at 9.62 min),^[^ ^12g^ ^]^ along with a minimal amount of Py‐Chal dimer, which was five times lower than the dimer formed at 440 nm and almost negligible given the sensitivity of MS. Importantly, no cross‐adducts were observed between the two reactive species, as confirmed in our previous publication,^[^ ^12c^ ^]^ which is key for the wavelength‐orthogonal network formation. Additionally, the absence of *o*MBA dimer formation at 440 nm indicates that there is no cross‐sensitization from the long‐wavelength‐active Py‐Chal to the short‐wavelength‐active *o*MBA. During printing, no lines were observed when the concentration of Py‐Chal in the dual resist was reduced from 25 to 13 wt.% (Figure S21, Supporting Information), further validating the absence of sensitization and indicating that only the Py‐Chal‐based network formed at 440 nm. In addition, the same fabrication window observed for **R1**‐based printing in a solvent mixture (acetophenone + propylene carbonate) as in propylene carbonate alone rules out an influence of acetophenone on the printing results (Table S3, Supporting Information). Micro‐FTIR was performed on the dual‐resist printed lines (Figure 4B) to directly probe their chemical compositions with spatial resolution, further confirming the wavelength‐orthogonality of the printed structures. The characteristic peaks of **S1**, as previously introduced in Figure 2E, are observed in lines printed at 440 nm from the dual resist (**D**_440_ _nm_), while the peaks corresponding to **S2** (Figure 3E) appear in **D**_320_ _nm_ (lines printed at 320 nm from the dual resist). Specifically, the peaks associated with **R1** and **S1**, such as those at 709 and 1658 cm^−1^, are present in **D**_440_ _nm_, but absent in **D**_320_ _nm_.^[^ ^20^, ^23^ ^]^ In contrast, the peaks corresponding to **R2** and **S2**, such as those at 778 and 1685 cm^−1^, are found in **D**_320_ _nm_, but not in **D**_440_ _nm_.^[^ ^24a^ ^]^

Furthermore, the IR spectrum of **D**_440_ _nm_ in the fingerprint region (600–1200 cm^−1^) closely resembles that of **R1** and **S1**, while the spectrum of **D**_320_ _nm_ is more similar to that of **R2** and **S2**. In addition, we observe that the highest peak of **R1**, **S1**, and **D**_440_ _nm_ appears at 1150 cm^−1^, whereas the highest peak in **D**_320_ _nm_ coincides with that of **R2** and **S2** at 1722 cm^−1^. When zooming into the 700–730 cm^−1^ region of **D**_320_ _nm_, the peak ratio of *I*_724 cm‐1_/*I*_709 cm‐1_ is close to 1, matching that of **R1**, but differing from **S1**, which lacks the 709 cm^−1^ signal. The above suggests the presence of **R1** in **D**_320_ _nm_, but not **S1**, as these two peaks are absent in **R2** and **S2**. The yellow color present in the lines printed at 320 nm further supports the presence of **R1**. All of the above demonstrates that the distinct materials were printed at two wavelengths.

To visualize the different materials, IR‐intensity signal ratio *I*_1685 cm−1_/*I*_1722 cm−1_ and *I*_1658 cm−1_/*I*_1722 cm−1_ were used to map the lines (Figure 4B). The resulting color values clearly distinguish the multi materials generated from a single print. The first three lines, printed at 440 nm, show a ratio of *I*_1685 cm−1_/*I*_1722 cm−1_ close to 0, while the ratio of *I*_1658 cm−1_/*I*_1722 cm−1_ is higher, corresponding to a Py‐Chal‐based network.^[^ ^23^ ^]^ In contrast, the last two lines, printed at 320 nm, exhibit the inverse trends, indicating an *o*MBA‐based network.^[^ ^24a^ ^]^ These distinct materials are also evident in the visualization of the *I*_1150 cm−1_/*I*_1722 cm−1_ ratio (Figure S22, Supporting Information).

#### 2.2.3 Degradable and Non‐Degradable Multi‐Material Structures

Significant changes in mechanical behavior were observed in **S1** and **S2** (Section 2.2.1.). Beyond the differences in mechanical properties, the truly remarkable novelty of our customized photoresist lies in its ability to extend the types of material properties addressable by multi‐material printing – from stiff/soft to degradable/non‐degradable – a capability that has not been previously demonstrated for macroscopically 3D printed constructs and would be much more challenging to achieve with semi‐orthogonal 3D printing approaches.

**Figure 5:**
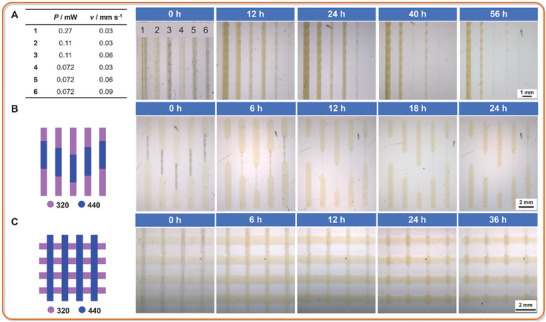
A) Lines printed from a dual‐resist system at 440 nm with varying laser power and printing speed, along with their degradation over time. B,C) bamboo‐ and fence‐shaped multi‐material structures printed at 320 and 440 nm in a single print, along with their degradation over time. The middle segments of (B) were printed at 440 nm with *P* = 0.072 mW (printing speed 0.09 mm s^−1^), while the outer segments were printed at 320 nm with *P* = 0.10 mW (printing speed 0.03 mm s^−1^). The vertical lines of C) were printed at 440 nm with *P* = 0.072 mW (printing speed 0.06 mm s^−1^), while the horizontal lines were printed at 320 nm with *P* = 0.10 mW (printing speed 0.03 mm s^−1^).

As discussed earlier, the reversibility of the [2 + 2] photo‐cycloaddition of Py‐Chal offers an opportunity for the degradation of printed structures. Before exploring degradable/non‐degradable multi‐material structures, we initially investigated the effect of writing conditions (power and printing speed) on degradation (**Figure** **5**A), taking advantage that only Py‐Chal is printed at 440 nm. Higher printing speed and lower power result in faster degradation, as evidenced by the complete degradation of line 6 after 12 h. Line 3 and 5 became significantly thinner after 24 h (from initially 528.0 to 328.5 µm after 12 h and 233.6 µm after 24 h, and from initially 480.9 to 283.1 µm after 12 h and 194.2 µm after 24 h, respectively) and completely disappeared after 40 h. Lines 1, 2, and 4, printed at the same speed, exhibited varying degradation rates: line 4 (initially 552.9 µm), printed at lower power, degraded more rapidly (to 392.7 µm after 12 h, 374.9 µm after 24 h, and 180.9 µm after 40 h, then completely disappearing after 56 h), while line 2 (initially 570.7 µm), although much thinner after 56 h (268.5 µm) than at 12 h (454.1 µm, then 434.1 µm after 24 h and 334.1 µm after 40 h), degraded more slowly than line 4, line 1 (from initially 650.7 µm, to 606.3 µm after 12 h, 602.2 µm after 24 h, to 545.5 µm after 40 h) remained intact even after 56 h (491.3 µm). Figure S24 (Supporting Information) has been provided to illustrate the degradation trends over time.

Note that after irradiation, all lines became more yellow indicating the reverse reaction from [2 + 2] dimer to monomer. Here, a UV‐B lamp with a peak emission at 313 nm was used (Figure S23, Supporting Information), as this wavelength favors the reverse reaction as discussed earlier.

Finally, we printed bamboo‐ and fence‐shaped multi‐material structures with two different wavelengths and subjected them to photodegradation studies. In the bamboo structures, the middle segments were printed at 440 nm (360.5 µm), while the other segments were printed at 320 nm (1003.9 µm) (Figure 5B). After 18 h, the middle segments almost disappeared (from 233.5 µm after 6 h to 139.6 µm after 12 h), whereas the segments printed at 320 nm remained intact. In the fence‐structure, the vertical lines were printed at 440 nm (389.3 µm), and the horizontal lines were printed at 320 nm (1072.8 µm) (Figure 5C). After 24 h, the vertical lines degraded significantly (from 373.6 µm after 6 h, 270.5 µm after 12 h to 147.4 µm after 24 h), with completion observed after 36 h. The joint part is more resistant to degradation due to the formation of an interpenetrated network. The slight remnants of the middle segments and vertical lines are likely due to adhesion between the glass substrate and the printed structures.^[^ ^27^ ^]^ A more pronounced yellowing was also present after irradiation, similar as in Figure 5A. These results confirm the successful printing of different materials using two wavelengths, with near complete degradation of structures printed at 440 nm further supporting the exclusive formation of the Py‐Chal dimer at this wavelength. In addition to the simple bamboo‐ and fence‐shaped multi‐material structures, we have fabricated a more complex cherry‐blossom‐like multi‐material structure and investigated its degradability under light irradiation. The G‐code for the cherry blossom and its trajectory are available in File S6 and Figure S25 (Supporting Information), respectively. In the cherry blossom structure, the center was printed at 440 nm, while the petal was printed at 320 nm (Figure S26, Supporting Information). After 12 h, the filament almost disappeared, whereas the petal printed at 320 nm remained intact. In addition, the center exhibited pronounced yellowing indicating degradation. After 24 h, the pistil and anther degraded significantly, however, these segments were more resistant to degradation due to their increased exposure from strongly overlapping pathways (Figure S25, Supporting Information). Thus, our wavelength‐orthogonal photochemistry enables the printing of degradable and non‐degradable materials simply by changing the color of light, a capability that our printer can achieve during the printing process by a simple change of monochromatic light color.

We acknowledge that the demonstrated structures are currently rather simple and can be better described as 2.5D rather than 3D. However, we argue that the presented 3D printing strategy has been demonstrated with a more readily available resin using multiple wavelengths (Figure 1C). Additionally, while our study focusses on single layer printing, it provides several advantages over patterning techniques: First, the process is simplified through direct fabrication. No coating steps and no mask are required, allowing for the free‐form design of structures. Additionally, we can fabricate structures with pre‐determined thicknesses, unlike thin‐layer patterning. Second, our approach offers parameter flexibility: The printing parameters can be adjusted dynamically during the process and allow access to a broad parameter range, freeing future resin development from such procedural limitations. Therefore, we argue that that our single layer printing builds the foundation for true color‐selective 3D printing from one resin.

## 3 Conclusion

We pioneer the truly pathway independent wavelength‐orthogonal printing for multi‐material structures enabled by the Mono LISA printer and a fully wavelength‐orthogonal photoresin. By integrating a monochromatic tunable laser with a custom‐designed 3D printing platform, Mono LISA enables accessing a broad wavelength and intensity range in a single print, allowing for the fabrication of complex, high resolution 2D and 3D objects. A dual photoresist system, combining a solely long‐wavelength active Py‐Chal‐based species and a solely short wavelength active *o*MBA‐based species, precisely gates the photochemical process, where the high‐energy wavelength is unable to active the low energy active species, leading to degradable and non‐degradable multi‐material objects. Our innovation enables the spatiotemporally resolved fabrication of disparate materials and properties from one resist as a function of only the color of light. Expansion of the spectrum of photochemical reactivity and the printer capabilities could unlock numerous possibilities for multi‐material AM and related applications.

Of course, several important material criteria will require further investigations in addition to these fundamental applications to translate them into applications. We have recently identified four main obstacles for multi‐wavelength printing,^[^ ^8a^ ^]^ which we have largely resolved in our current work via a fundamental proof of concept. Nonetheless, each criterium will require additional attention in the future.

First and foremost, we herein present a hardware and software solution – Mono LISA – that enables materials photochemistry with unprecedented precision and tuneability regarding irradiation wavelength and intensity. Despite the broad wavelength and intensity tunability of our printer, we recognize that the cost of tunable lasers remains high. However, we anticipate that wavelength‐tunable laser systems will become more readily available, like other laser technologies. Of course, this progress will require collaboration with laser physicists and engineers.

We further demonstrate a first example for a multi‐material resin with true wavelength orthogonal resolution: Printing of either resin without the second resin has been achieved. Solvent is currently required to obtain liquid resins with the desired viscosity due to the solubility and viscosity of the utilized chromophore‐containing prepolymers. The solvent also serves to dilute the long‐wavelength‐active chromophore‐containing prepolymers, limiting their reactivity in the UV region. Additionally, the incorporation of the other prepolymers reduces the solvent content in the dual‐resist system, maintaining a concentration of both prepolymers comparable to that in the single‐resist system. Nonetheless, the inclusion of solvent may limit the achievable thermomechanical performance, such as inducing volumetric shrinkage. In the future, advanced prepolymers or small molecule designs, such as multi‐arm chromophores, and increased printing temperatures may resolve these issues.

In addition to the solvent, the scalability of the photoresists plays a critical role in translating them into applications. The cost and availability of the starting materials, as well as the feasibility of the synthesis, should be given significant consideration.

It is highly interesting and encouraging that we did not observe cross‐sensitization for the photoresins investigated herein and we successfully achieved fully wavelength orthogonal printing. However, residual UV reactivity was still observed in the reported long‐wavelength‐active Py‐Chal‐based photoresist. To address this, we limited the photoreactivity of Py‐Chal by decreasing the concentration of the Py‐chal‐based prepolymer and controlling the irradiation intensity. The cycloaddition‐reversion reaction in the UV regime was also shown to support orthogonal printing. While limiting the photoreactivity contributed to the success of the first fully orthogonal printing, it is important to note that such limitations may significantly reduce the quantum yield and strongly impact printing speeds in other systems. To overcome these challenges, it is vital to move beyond the UV range, where the reactivity of most photoactive compounds overlaps, and achieving λ‐orthogonality is highly challenging. Thus, designing new chromophores in the visible light range is essential. In addition, photochemical action plot studies are needed to identify the reactivities and λ‐orthogonal windows of the chromophores.

## Conflict of Interest

The authors declare no conflict of interest.

## Supporting information



Supporting Information

Supplemental File 1

Supplemental File 2

Supplemental File 3

Supplemental File 4

Supplemental File 5

Supplemental File 6

Supplemental File 7

## Data Availability

The data that support the findings of this study are available from the corresponding author upon reasonable request.
