# Supplementary material for: Two Material Properties from One Wavelength‐Orthogonal Photoresin Enabled by a Monochromatic Laser Integrated Stereolithographic Apparatus (Mono LISA)
Source: Adv Mater. 2025 Feb 17;37(13):2419639. doi: 10.1002/adma.202419639 (PMC11962704; doi:10.1002/adma.202419639)
Supplement: Supplementary file 1 — Supporting Information [file ADMA-37-2419639-s003.pdf]

ADVANCED MATERIALS

Supporting Information

for *Adv. Mater.*, DOI 10.1002/adma.202419639

Two Material Properties from One Wavelength-Orthogonal Photoresin Enabled by a Monochromatic Laser Integrated Stereolithographic Apparatus (Mono LISA)

Xingyu Wu, Katharina Ehrmann*, Ching Thye Gan, Benjamin Leuschel, Fred Pashley-Johnson and Christopher Barner-Kowollik**

Supporting Information

Two Material Properties from One Wavelength-Orthogonal Photoresin Enabled by a Monochromatic Laser Integrated Stereolithographic Apparatus (Mono LISA)

Xingyu Wu[‡] Katharina Ehrmann[‡]* Ching Thye Gan, Benjamin Leuschel, Fred Pashley-Johnson, Christopher Barner-Kowollik**

Table of Contents

1. Materials……………………………………………………………………………….3
2. Instrumentation……………………………………………………………………...3
   2.1. Flash Chromatography……………………………………………………………3
   2.2. Nuclear Magnetic Resonance (NMR)……………………………………………..4
   2.3. Liquid Chromatography Mass Spectroscopy (LC-MS) ………………………….4
   2.4. THF Size Exclusion Chromatography (THF-SEC) ………………………………4
   2.5. UV-Vis spectroscopy……………………………………………………………...5
   2.6. Optical Microscopy………………………………………………………………..5
   2.7. IR Microscopy……………………………………………………………………..5
   2.8. Nanoindentation……………………………………………………………………5
3. Mono LISA……………………………………………………………………………6
   3.1. Setup……………………………………………………………………………….6
   3.2. ZnTPP-Based Multi-Color 2D and 3D Printing…………………………………..8
   3.3. Feature Sizes of Lines Printed at Different Wavelengths…………………………9
4. Pathway Independent Wavelength-orthogonal Printing……………………………..11
   4.1. Synthetic Procedures……………………………………………………………11
      4.1.1. Synthesis of Poly-(Py-Chal)$_n$-*co*-TEGMA$_m$……………………………………11
      4.1.2. Synthesis of Poly-(*o*MBA)$_n$-*co*-MMA$_m$…………………………………...15
   4.2. Photochemical Action Plot………………………………………………………17
   4.3. R1- and R2-based Printing……..……………………………………………………17
   4.4. Concentration-dependence of the R1-based Photoresist on Printing Behavior ………19
   4.5. Selectivity Windows………………………………………………………………20
   4.6. Irridiation of Py-Chal and *o*MBA Model Compound Mixture at 440 and 320 nm.……22
   4.7. Lines Printed from Dual-resist System with Low Concentration of R1………………24
   4.8. R1-based Printing with Different Solvents…………………………...………………24
   4.9. Chemical Composition Mapping with FTIR………………………………………24
   4.10. Degradation of Multi-material Structures…………………………………………25
   References…………………………………………………………………………27

1. Materials

All chemicals and solvents were used as received from the supplier without further purification, unless stated otherwise.

4'-Hydroxy-3'-methoxyacetophenone (acetovanillone) (98 %, Sigma-Aldrich), ethyl 4-bromobutyrate (98 %, Combi Blocks), 1-pyrenecarboxaldehyde (98 %, Combi Blocks), potassium carbonate (anhydrous, Chemsupply), sodium hydroxide (pellets, Chemsupply), sulfuric acid (98 %, Chemsupply), triethylene glycol methyl ether methacrylate (TEGMA, 93 %, Sigma-Aldrich, after passing through a short plug of basic alumina), azobisisobutyronitrile (AIBN, Sigma-Aldrich, recrystallised), dodecyl mercaptan (DDM, ≥98%, Sigma-Aldrich), methyl methacrylate (MMA, 99 %, Sigma-Aldrich, after passing through a short plug of basic alumina), 2-hydroxyethyl methacrylate, (HEMA, 97 %, Sigma-Aldrich), 18-crown-6 (99%, Fisher Scientific), dichloromethane (Fisher Scientific), methyl 4-(bromomethyl) benzoate (98 %, Sigma-Aldrich), acetone (Fisher Scientific), methanol (Fisher Scientific), potassium persulfate ($K_2S_2O_8$, ≥99.0%, Sigma-Aldrich), Copper (II) sulphate pentahydrate ($CuSO_4{\cdot}5H_2O$, ≥98.0%, VMR), acetonitrile (anhydrous, Thermo Fischer), hexane (Fisher Scientific), ethyl acetate (Fisher Scientific), basic alumina oxide (VWR), $AlCl_3$ (Sigma-Aldrich), hydrochloric acid (HCl), sodium sulfate (anhydrous, ≥99.0%, Fisher Scientific, *N,N'*-dicyclohexylcarbodiimide (DCC, 99%, Sigma-Aldrich), 4-dimethylaminopyridine (DMAP, ≥99 %, Sigma-Aldrich), *N,N'*-dimethylformamide (DMF, anhydrous, Fisher Scientific), *N*-(3-dimethylaminopropyl)-*N'*-ethyl carbodiimide hydrochloride (EDC, ≥98.0%, Sigma-Aldrich), cyclohexane (Fisher Scientific), ethanol (Fisher Scientific), propylene carbonate (anhydrous, Fisher Scientific), acetophenone (≥99.0%, Sigma-Aldrich), 5,10,15,20-tetraphenyl-21*H*,23*H*-porphine zinc (ZnTPP, Sigma-Aldrich), 2-propanol (Fisher Scientific), dimethyl sulfoxide (DMSO, Fisher Scientific). All deuterated solvents were purchased from Cambridge Isotope Laboratories.

2. Instrumentation

2.1. Flash Chromatography

Flash chromatography was performed on an Interchim XS420+ flash chromatography system consisting of an SP-in-line filter 20-µm, an UV-VIS detector (200-800 nm) and a SofTA Model 400 ELSD (55 °C drift tube temperature, 25 °C spray chamber temperature, filter 5, EDR gain mode) connected via a flow splitter (Interchim Split ELSD F04590). The separations were

performed using an Interchim dry load column and an Interchim Puriflash Silica HP 30 μm column.

2.2. Nuclear Magnetic Resonance (NMR)

^{1}H-, ^{13}C-NMR and COSY spectra were recorded on a Bruker System 600 Ascend LH, equipped with a BBO-Probe (5 mm) with z-gradient (^{1}H: 600.13 MHz, ^{13}C 150.90 MHz). All measurements were carried out in deuterated solvents. Resonances are reported in parts per million (ppm) relative to residual solvent protons. The measured coupling constants were calculated in Hertz (Hz). Spectra were analyzed with MestReNova 11.0.

2.3. Liquid Chromatography Mass Spectroscopy (LC-MS)

LC-MS measurements were performed on a Dionex UltiMate 3000 UHPLC system consisting of a quaternary pump (LPG 3400RS), autosampler (WPS 3000TRS) and column oven (TCC 3000). A 10 μL aliquot of sample was injected onto a C18 HPLC column (Phenomenex Luna 5μm, 100 Å, 250 × 2.0 mm) maintained at 40 °C. Mobile phase A was 5 mM ammonium acetate in water and mobile phase B was acetonitrile. The combined flow rate was maintained at 0.4 mL min^{-1}. After an initial 0.6 min isocratic period at 20% B, the gradient was increased to 95% B over 7 min, then held at 95% B for a further 3 min before returning to 20% B. The eluate was directed to a UV diode array detector (DAD 3000, Dionex) and subsequently into the heated electrospray ionisation source of a Q Exactive Plus Biopharma high-resolution Orbitrap mass spectrometer (Thermo Fisher Scientific, Bremen, Germany). The spray voltage was set to 3.0 kV, and the sheath and auxiliary gas (N_2) flow rates were set to 30 and 10 (dimensionless arbitrary units), respectively. The capillary temperature was set to 320 °C, the S-lens RF level was set to 60, and the auxiliary gas heater temperature was set to 150 °C. Spectra were acquired at a nominal mass resolving power of 70,000 (defined at *m/z* 200).

2.4. THF Size Exclusion Chromatography (THF-SEC)

The SEC measurements were conducted on a PSS SECurity system consisting of a PSS SECurity Degasser, PSS SECurity TCC6000 Column Oven (35 °C), PSS SDV Column Set (8 x 150 mm 5 µm Precolumn, 8 x 300 mm 5 µm Analytical Columns, 100000 Å, 1000 Å and 100 Å) and an Agilent 1260 Infinity Isocratic Pump, Agilent 1260 Infinity Standard Autosampler, Agilent 1260 Infinity Diode Array and Multiple Wavelength Detector (A: 254 nm, B: 360 nm), Agilent 1260 Infinity Refractive Index Detector (35 °C). HPLC grade THF, stabilized with BHT, is used as eluent at a flow rate of 1 mL·min^{-1}. Narrow disperse linear poly(styrene) (M_n:

266 g·mol^{-1} to 2.52x10^{6} g·mol^{-1}) and poly(methyl methacrylate) (M_n: 202 g·mol^{-1} to 2.2x10^{6} g·mol^{-1}) standards (PSS ReadyCal) were used as calibrants. All samples were passed over 0.22 µm PTFE membrane filters. Molecular weight and dispersity analysis was performed in PSS WinGPC UniChrom software (version 8.2).

2.5. UV-Vis Spectroscopy

UV-Vis spectra were recorded on a dual-beam Cary 60 UV-Vis spectrophotometer. The path length was always set to 1 cm. Baseline corrections were performed with the same cuvette and solvent used for the sample data acquisition. The scan rate was 300 nm min^{-1} with a data interval of 0.5 nm, and an averaging time of 0.1 seconds. Data was collected without corrections.

2.6. Optical Microscopy

Light microscope images were carried out using a Leica M125 Stereo Microscope equipped with LED white light source. Samples were placed on the window and imaged at various magnifications with transmitted light. Images were acquired via Leica Application Suite (LAS) X software.

3D images were acquired by digital optical microscope DSX1000 (Olympus) with a 10× long working distance objective. The feature sizes of structures were analyzed with DSX software.

2.7. IR Microscopy

Infrared spectra were recorded with a Nicolet iS50 Fourier Transform Infrared (FTIR) spectrometer in combination with a Continuum IR microscope equipped with a Ge attenuated total reflectance (ATR) objective and a liquid N_2 cooled mercury-cadmium-telluride (MCT) detector. The spectra were recorded at 4 cm^{-1} resolution in the range of 4000 to 650 cm^{-1} after accumulating 64 scans.

The IR microscope has a motorized stage, which allows samples to be mapped in the X and Y directions. For mapping, each spectrum composed of 4 scans with a resolution of 8 cm^{-1} was collected.

Analysis was performed using OMNIC Atlµs software.

2.8. Nanoindentation

Nanoindentation tests were performed using a Hysitron TI950 Nanoindenter. A tip with the Berkovich geometry was used to perform all measurements. All tests were performed under

load control. The *o*MBA network was indented to 800 µN with a loading rate of 20 µNs^{-1} and was unloaded at a rate of -50 µNs^{-1}. The PyChal network was indented to a load of 70 µN with a loading rate of 5 µNs^{-1} and an unloading rate of -20 µNs^{-1}. Both methods had a 60 s period at peak load to allow the network to relax, which was assessed by inspecting the time vs displacement curves presented in **Figure S14**. The curves were analysed in TriboScan 10.2.1.0 and fitted with the Oliver-Phar method using the unloading curve, excluding the first 5% and last 25%. Each material was indented 16 times, and the mean value taken ± standard error.

3. Mono LISA

3.1. Setup

All components of the 3D printing system are mounted on an optical table. As shown in Scheme S1, an Opotek Opolette 355 OPO wavelength-tunable laser producing 7 ns, 20 Hz pulses with a flattop signal profile and a beam output size of 4 mm (①) serves as the light source. The output beam reflects through UV-enhanced aluminum mirrors (③ and ④, avg. reflectance > 90%) and subsequently passes through an electronic shutter (⑤). It further passes a spherical focusing lens (⑥, f = 300 mm) mounted on a long-travel stage and directs to a 3-axis stage (⑧, Thorlabs motorized translation stage PT3-Z29 with three K-Cube DC Servo Motor Controllers) using a UV silica right angle prism (⑦, 185 nm - 2.1 µm). The power meter (②) is positioned between the laser and the first mirror to measure all powers for printing, ensuring both convenience and safety. It is important to note that power adjustments and verifications during printing must be performed without moving the sample. Based on these considerations, we believe that measuring power directly at the beam output is the optimal approach. The resin tank is placed under the X, Y and Z stage by a tank holder and the build platform with a movable bar is mounted on the X, Y and Z stage. Photographs of the printing platform including the 3-axis (X, Y and Z) stage, build platform, resin tank and tank holder as well as models of these three components, are shown in Figure S1. The movement of the long-travel stage, the X, Y and Z stage as well as the shutter (on-off) is controlled by our customized software (Supplementary file 1), programmed in LabView. Upon importing G-code files containing the coordinates and travel speed (Supplementary files 2-5), the software processes the files to precisely control both the printing trajectory and speed.

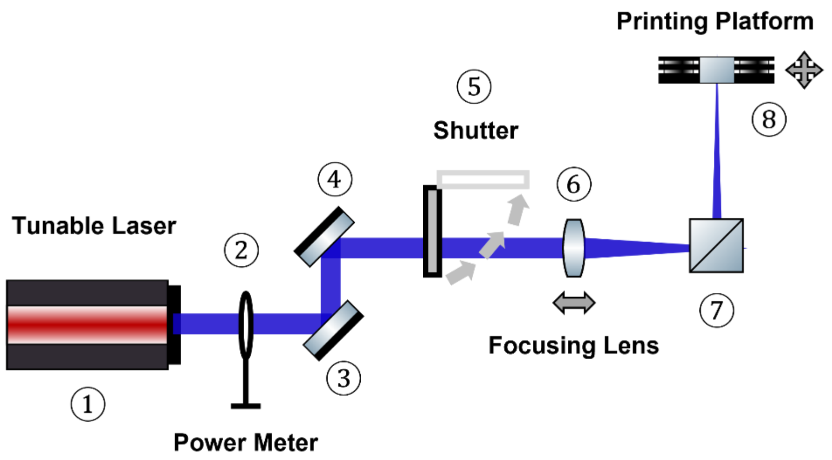

Scheme S1. Schematic of the Mono LISA printer, showing the beam path and various components.

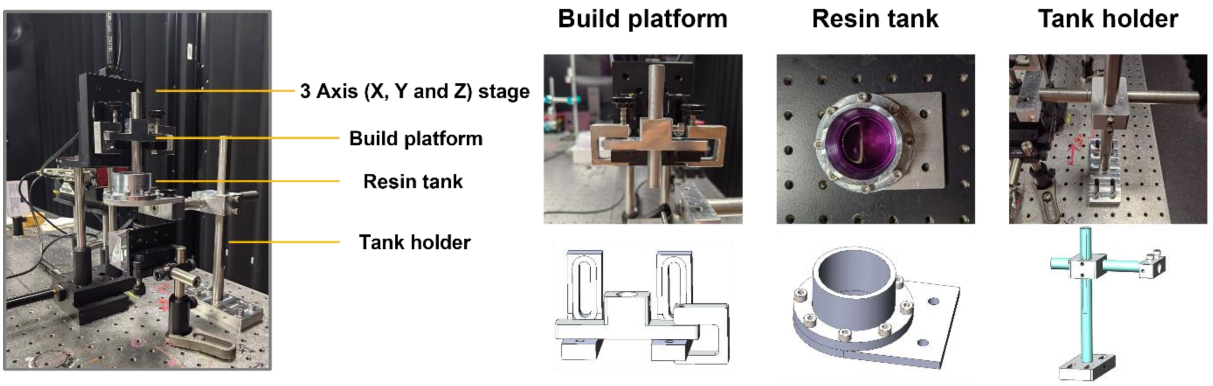

Figure S1. Photographs of the wavelength-tunable laser printing platform and the CAD models of three components.

The LabView software is used to establish communication with each of the motors' controllers of the 3-axis (X, Y and Z) stage and the long-travel stage. Once communication is established, LabView can automate sending the X, Y and Z coordinates to the controllers of the 3-axis stage, allowing the three motors to move at the same time, as well as defining the velocity for each axis. Furthermore, in order to automate a series of commands to enable 2D and 3D printing, the LabView program is written to accept the widely used G-code file format. G-code files can be generated using commercially available software, such as UltiMaker Cura (v. 5.6.0). Here it is worth noting that our LabView software allows us to define the velocity. However, the current velocity is limited by the mechanical stage which has a maximum speed of 2.5 mm s^{-1}. Additionally, as with most mechanical stages, it has limitations in its control at the start and end of the stage's movement. Higher printing speed can lead to extended laser travel distances

during the acceleration and deceleration phases, causing uneven resin exposure. These factors must be taken into account when selecting the printing speed.

3.2. ZnTPP-Based Multi-Color 2D and 3D Printing

A ZnTPP-based photoresist for 2D printing was prepared by mixing ZnTPP (23.58 mg) with PETA (12.20 g) and DMF (260 mg).[1] After placing the glass sample holder onto the X, Y and Z stage, the photoresist was directly deposited onto glass slide for printing. The G-code files for lines and interlocking rings were generated with UltiMaker Cura (v. 5.6.0). The beam, by passing through the focusing lens, was focused onto the surface of the glass substrate by adjusting the long-travel stage. The objects were subsequently printed by controlling the movement of the stage in the X and Y directions with the software.

The printing speed for all ZnTPP-based 2D printing was maintained at 0.1 mm s^{-1}. The laser powers used for the four rings in one row were 0.77 ± 0.025 mW at 360 nm, 1.68 ± 0.087 mW at 430 nm, 2.24 ± 0.078 mW at 500 nm and 2.90 ± 0.087 mW at 565 nm. For the five rings in two rows, the reported laser powers were applied from 360 nm to 565 nm and 3.77 ± 0.098 mW were applied at 600 nm.

The photoresist for 3D printing consists of ZnTPP (0.37 mg), PETA (12.20 g), and DMF (7.55 mg) for printing at 360 nm and 430 nm, and of ZnTPP (2.59 mg), PETA (12.20 g), and DMF (52.85 mg) for printing at 500 nm, adjusting for the varying initiation efficiency of ZnTPP at the different wavelengths. The G-code file for a pyramid was also generated with Cura. In contrast to 2D printing, the beam was focused on the surface of the resin tank's film and the objects were printed by lifting the build platform via the Z-stage movement (step size 0.2 mm), in addition to the movement in the X and Y directions.

The printing speed for all ZnTPP-based 3D printing was maintained at 0.1 mm s^{-1}. In 3D printing, laser powers of 1.05 ± 0.035 mW, 1.76 ± 0.085 mW and 3.42 ± 0.077 mW were applied at 360 nm, 430 nm and 500 nm, respectively. After laser exposure, the objects were developed with 2-propanol.

The G-code files for the interlocking rings and the pyramid are provided in Supplementary files 2-4. Figure S2 illustrates the trajectory and coordinates in the G-codes for each structure.

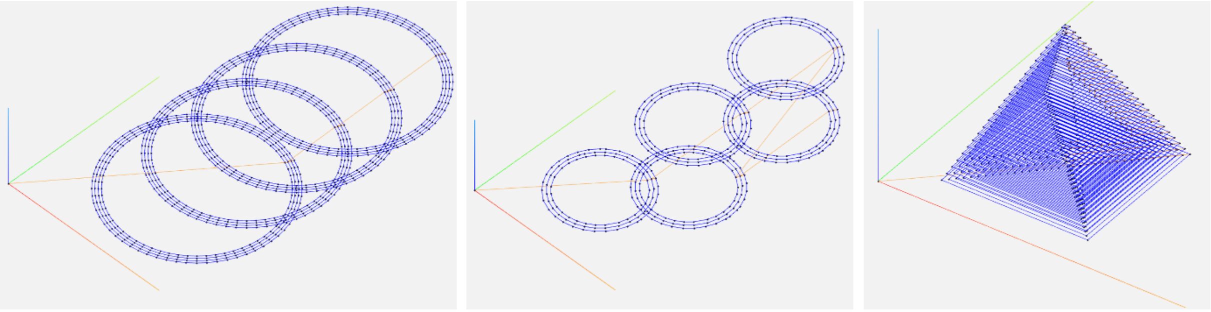

Figure S2. Images illustrating the trajectory of the 3-axis (X, Y and Z) stage for the interlocking rings and the pyramid, generated from the G-code files to obtain the corresponding structures. The blue lines represent the trajectory when the shutter is on (i.e., resin exposure to the laser) with blue dots indicating the coordinates. The orange lines show the trajectory when the shutter is off (i.e., resin is not exposed to the laser).

3.3. Feature Sizes of Lines Printed at Different Wavelengths

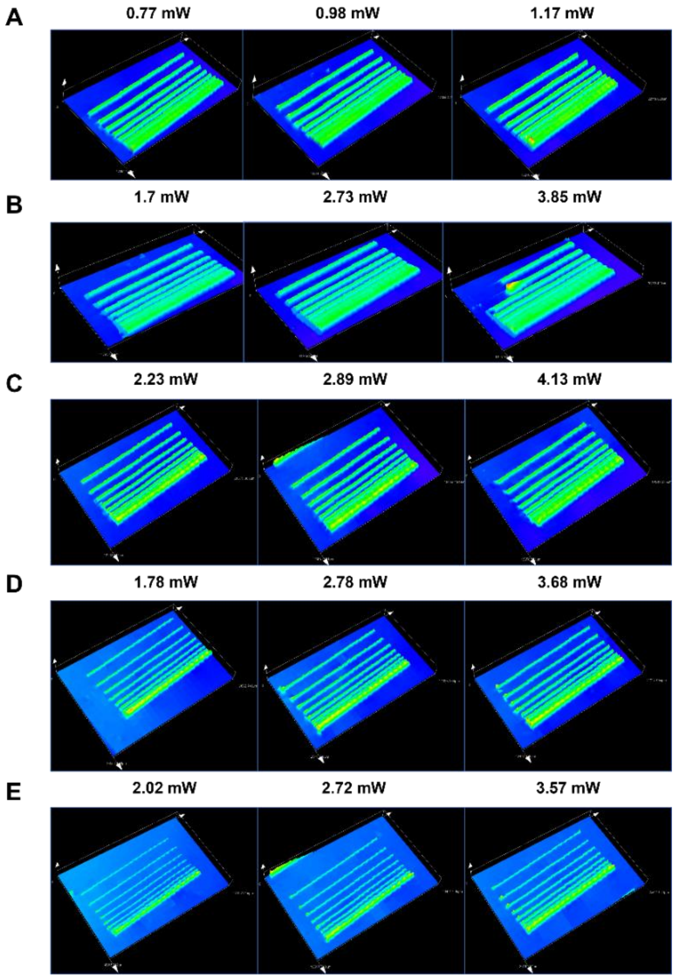

Figure S3. Optical microscopy images of lines printed at (A) 360 nm, (B) 430 nm, (C) 500 nm, (D) 565 nm, and (E) 600 nm at varying laser powers using a ZnTPP-based photoresist.

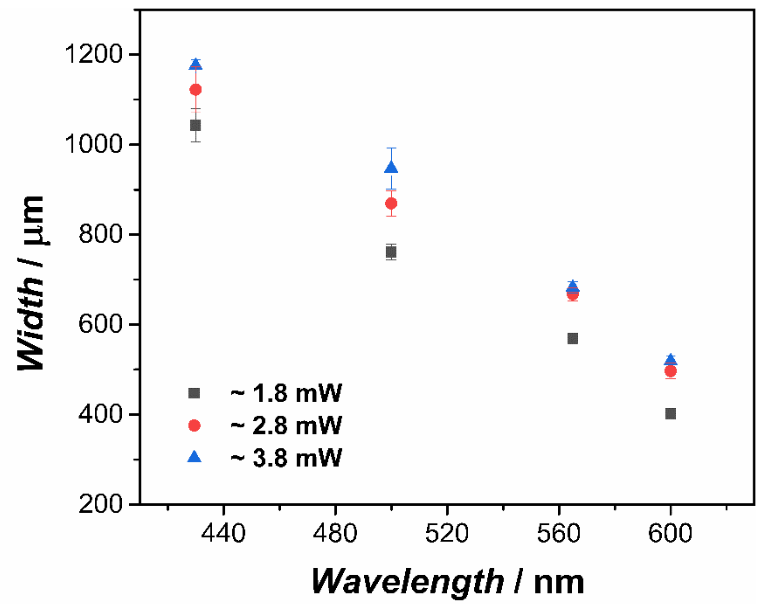

Figure S4. Line widths, measured from the images in Figure S3, plotted against wavelength.

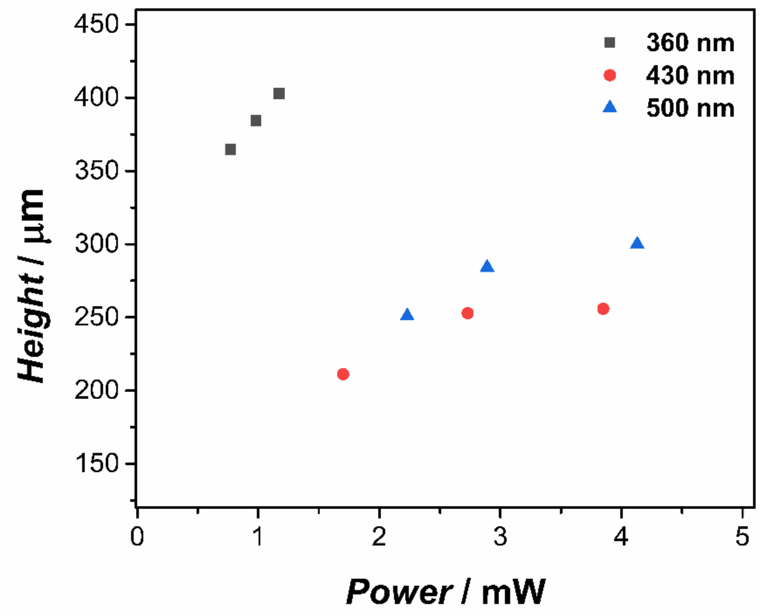

Figure S5. Line heights at three wavelengths, measured from the images in Figure S3, plotted against power.

As shown in Figure S5, the height of the lines at each wavelength increases with power. At 360 nm, a perfect linear relationship is observed, while at 430 nm and 500 nm, the increase in line height slows towards the end, likely approaching height saturation due to the high power. As the laser's power at 360 nm is limited, it was not possible to obtain lines fabricated at powers higher than 1.2 mW. Since the minimum heights exceed 200 µm, we have selected 200 µm for the Z-step distance in 3D printing and reduced the concentration of ZnTPP to achieve a higher penetration depth.

At each wavelength, the laser was operated at maximum power, delivering 10 single-shot pulses targeted at photosensitive paper. The resulting marks were imaged using an optical microscope (Leica M125), and the micrographs were analyzed in ImageJ to determine the beam area.

Table S1. The beam sizes and intensities at the printing site correlated with the output power.

Wavelength / nm	Power at beam output / mW	Beam size at printing site / mm^2	Intensity at printing site / mW cm^{-2}
**360**	0.77	0.847	42.73
	0.98		54.38
	1.17		64.92
**430**	1.7	1.040	70.29
	2.73		112.88
	3.85		159.18
**500**	2.23	1.200	74.33
	2.89		96.33
	3.42		114.00
	4.13		137.67
**565**	1.78	1.070	64.88
	2.78		101.33
	3.68		134.13
**600**	2.02	0.853	73.41
	2.72		98.85
	3.57		129.74

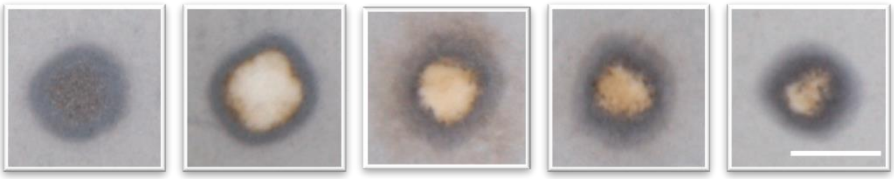

Figure S6. Spatial mapping of the laser spot at 360, 430, 500, 565 and 600 nm from left to right (the size of the scale bar is 1 mm).

4. Pathway Independent Wavelength-Orthogonal Printing

4.1. Synthetic Procedures

4.1.1. Synthesis of Poly-(Py-Chal)$_n$-co-TEGMA$_m$

To obtain the Py-Chal-containing prepolymer, Py-Chal and the Py-Chal-based methacrylate monomer (Py-Chal-HEMA) was synthesized (Scheme S2) and subsequently the monomer employed as comonomer in a radical polymerization (Scheme S3).

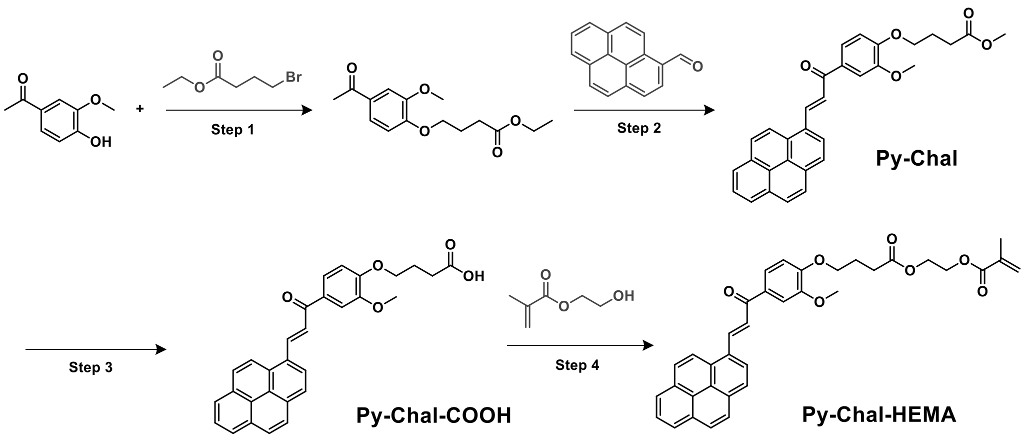

Scheme S2. Synthesis of Py-Chal and Py-Chal-HEMA.

Steps 1 to 3 of the Py-Chal monomer synthesis were performed according to literature procedures.[2]

Step 4: Py-Chal-HEMA was synthesized in an adaptation of literature procedures.[3] Py-Chal-COOH (2.0 g, 4.3 mmol, 1 eq.), 2-hydroxyethylmethacrylate (0.6 mL, 5.2 mmol, 1.2 eq.) and 4-dimethylaminopyridine (DMAP, 0.1 g, 0.7 mmol, 0.2 eq.) were dissolved in DMF (40 mL) and cooled in an ice bath. After adding *N*-(3-dimethylaminopropyl)-*N'*-ethyl carbodiimide hydrochloride (EDC, 1.8 g, 9.5 mmol, 2.2 eq.), the solution was allowed to warm to ambient temperature and stirred overnight. The crude reaction mixture was adsorbed onto celite and purified using silica gel column chromatography yielding 1.5 g (60%) of a yellow powder.

^{1}H NMR (600 MHz, DMSO-d_6) δ / ppm = 8.90 – 8.76 (m, 2H), 8.63 (d, J = 9.4 Hz, 1H), 8.43 – 8.32 (m, 4H), 8.31 – 8.23 (m, 3H), 8.13 (t, J = 7.6 Hz, 1H), 8.01 (dd, J = 8.5, 2.0 Hz, 1H), 7.72 (d, J = 2.0 Hz, 1H), 7.14 (d, J = 8.5 Hz, 1H), 6.02 (dq, J = 2.0, 1.0 Hz, 1H), 5.67 (p, J = 1.6 Hz, 1H), 4.31 (s, 4H), 4.13 (t, J = 6.4 Hz, 2H), 3.91 (s, 3H), 2.57 – 2.50 (m, 2H), 2.03 (p, J = 6.7 Hz, 2H), 1.86 (dd, J = 1.6, 1.0 Hz, 3H).

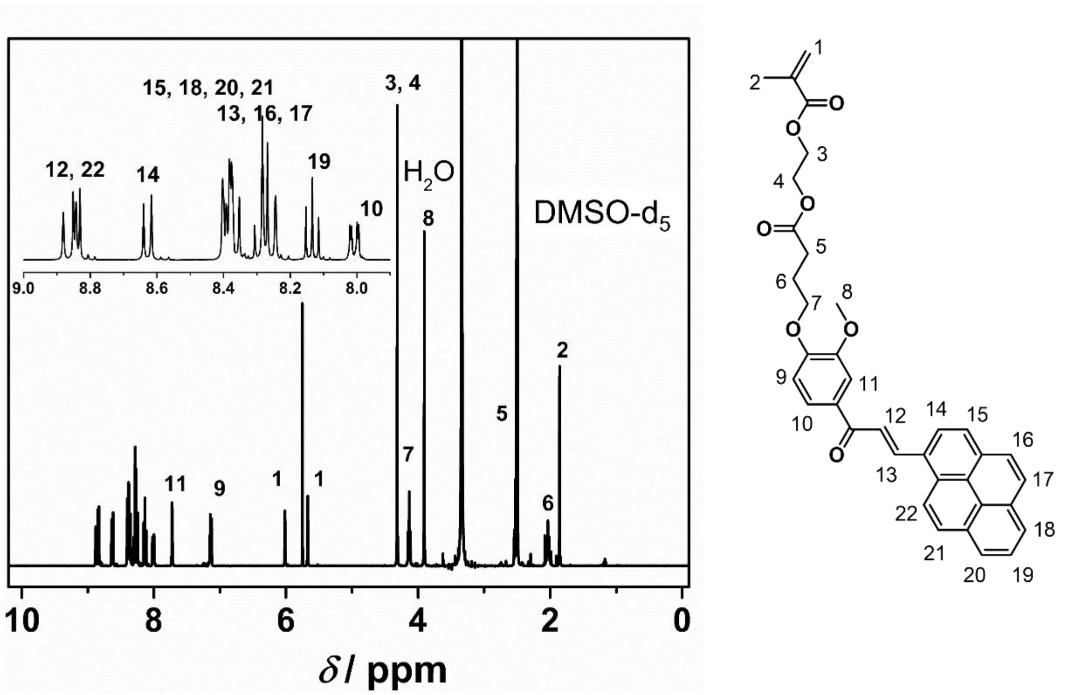

Figure S7. ^{1}H NMR spectrum of Py-Chal-HEMA in DMSO-d_6.

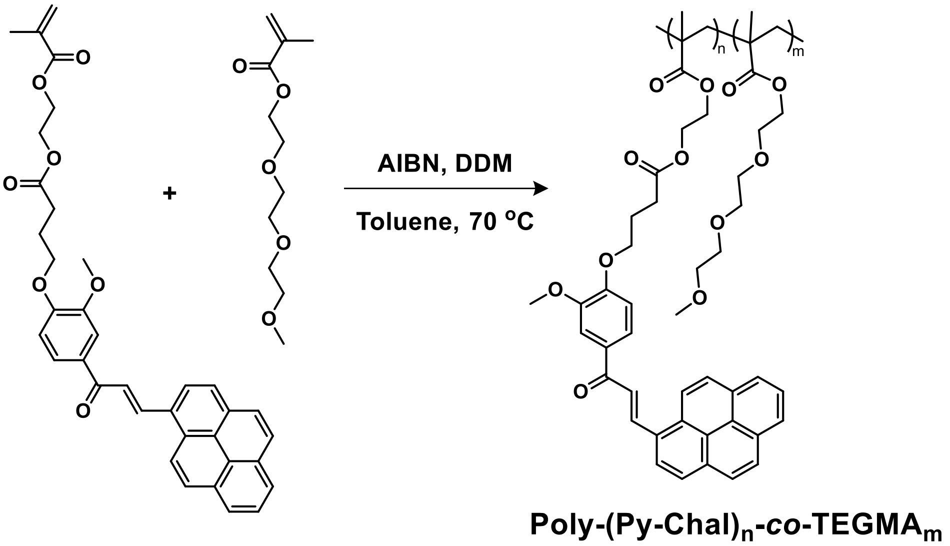

Poly-(Py-Chal)$_n$-*co*-TEGMA$_m$

Scheme S3. Synthesis of **R1** Poly-(Py-Chal)$_n$-*co*-TEGMA$_m$.

R1: Py-Chal-HEMA (1.0 g, 1.7 mmol), triethylene glycol methyl ether methacrylate (TEGMA, 2.2 mL, 9.7 mmol), dodecyl mercaptan (DDM, 125 µL, 0.52 mmol) and azobisisobutyronitrile (AIBN, 37 mg, 0.23 mmol) were combined in 30 mL toluene. The solution was degassed for 15 minutes and then stirred overnight at 70 °C. The reaction was cooled to ambient temperature and the toluene removed *in vacuo*. The residue was redissolved in a minimal amount of THF and purified by precipitation into cold methanol (x3) and cold pentane (x2). The polymer was recovered as a yellow resin.

^{1}H NMR (600 MHz, DMSO-d_6) δ / ppm = 8.89 – 8.64 (m, CH-12), 8.64 – 7.60 (m, CH-10-11, 13-22), 7.17 – 6.94 (m, CH-9), 4.34 – 4.19 (m, CH_2-3, 4), 4.19 – 3.98 (m, CH_2-7, 23), 3.96 – 3.76 (m, CH_3-8), 3.62 – 3.35 (m, CH_2-24-28), 3.27 – 3.05 (m, CH_3-29), 2.53 (m, CH_2-5) 2.07 (m, CH_2-6), 1.96 – 0.60 (m, polymer backbone-1, 2).

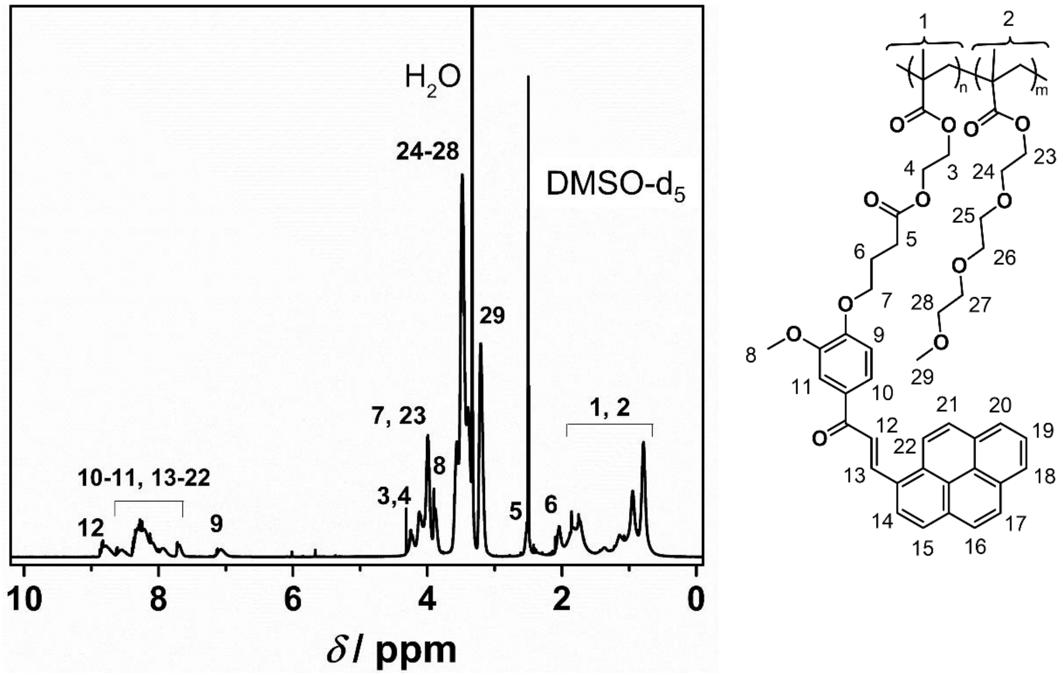

Figure S8. ^{1}H NMR spectrum of **R1** in DMSO-d_6.

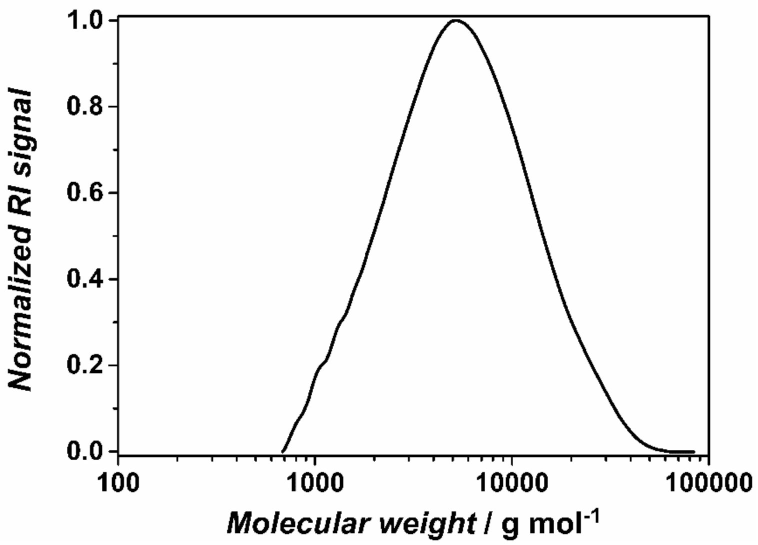

Figure S9. SEC trace of **R1** in THF using a PMMA calibration.

4.1.2. Synthesis of Poly-(oMBA)$_n$-co-MMA$_m$

For the synthesis of the *o*MBA-containing prepolymer, *o*MBA and the *o*MBA-methacrylate monomer (*o*MBA-HEMA) was synthesized (Scheme S4) and subsequently the monomer employed as comonomer in a radical polymerization (Scheme S5).

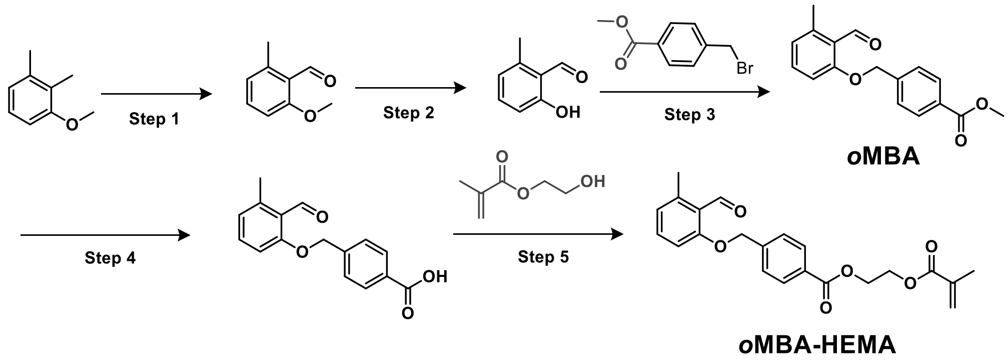

Scheme S4. Synthesis of *o*MBA and *o*MBA-HEMA.

For the synthesis of *o*MBA-HEMA, **steps 1 to 5** were performed according to literature procedures.[4]

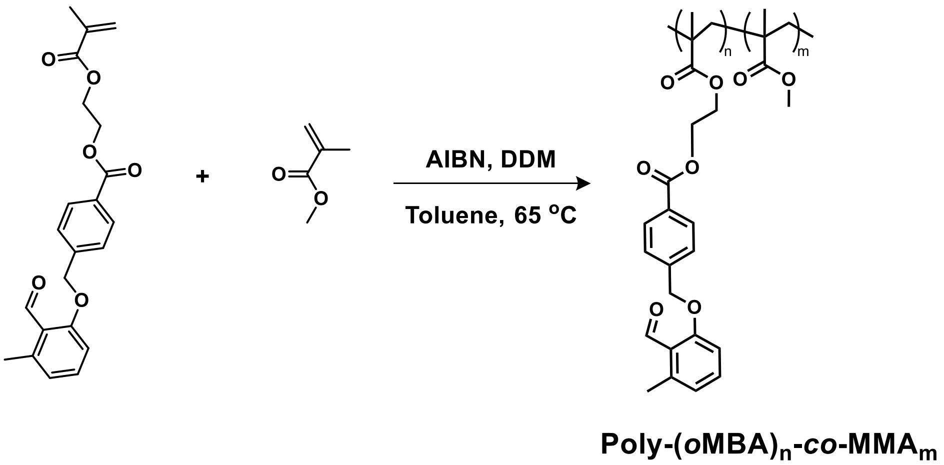

Scheme S5. Synthesis of **R2** (Poly-(*o*MBA)$_n$-*co*-MMA$_m$.

R2: Methyl methacrylate (1.11 mL, 10.50 mmol, 40 eq.), *o*MBA-HEMA (1.0 g, 2.60 mmol, 10 eq.) and AIBN (43.0 mg, 0.26 mmol, 1 eq.), DDM (62.6 µL, 0.26 mmol, 1 eq.) were dissolved in 30 mL toluene. The solution was purged with nitrogen for 15 minutes, the flask sealed, placed in an oil bath at 65 °C and removed after 24 h. The flask was cooled with ice to stop the reaction. The mixture was precipitated twice out of cold methanol + 10% H_2O and the precipitate was dried in a vacuum oven over night at 40 °C.

¹H-NMR (600 MHz, $CDCl_3$) δ / ppm = 10.74 (bs, CHO-14), 8.16 – 7.97 (m, CH-5, 7), 7.59 – 7.46 (m, CH-6, 8), 7.41 – 7.31 (m, CH-11), 6.96 – 6.77 (m, CH-10, 12), 5.33 – 5.10 (m, CH_2-9), 4.60 – 4.16 (m, CH_2-3, 4), 3.57 (bs, CH_3-15), 2.58 (bs, CH_3-13), 2.10 – 0.71 (m, polymer backbone-1, 2).

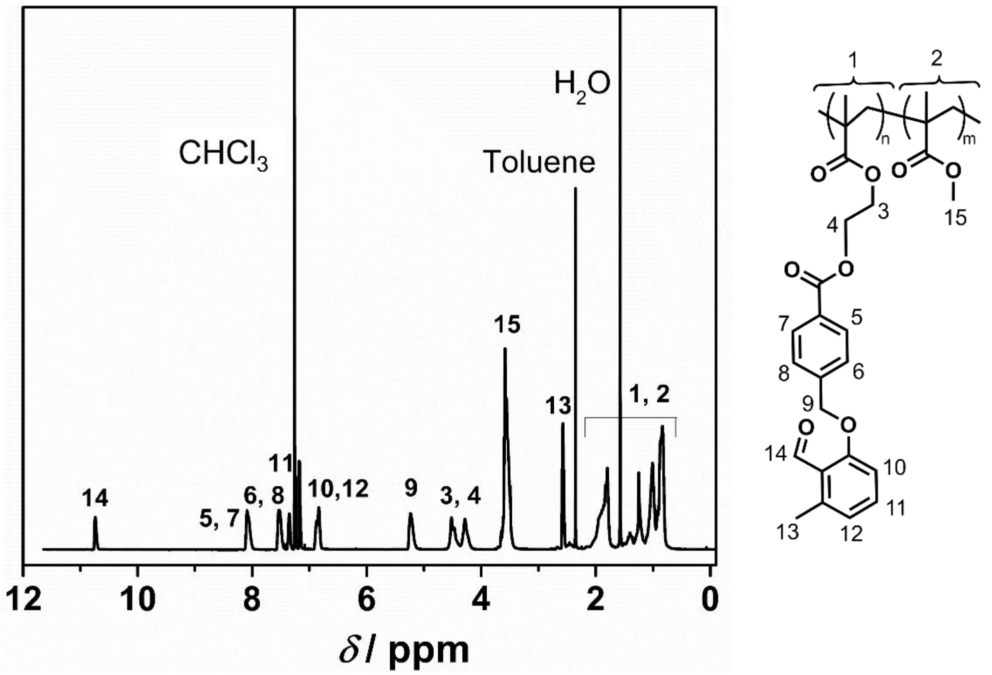

Figure S10. ¹H NMR spectrum of **R2** in $CDCl_3$.

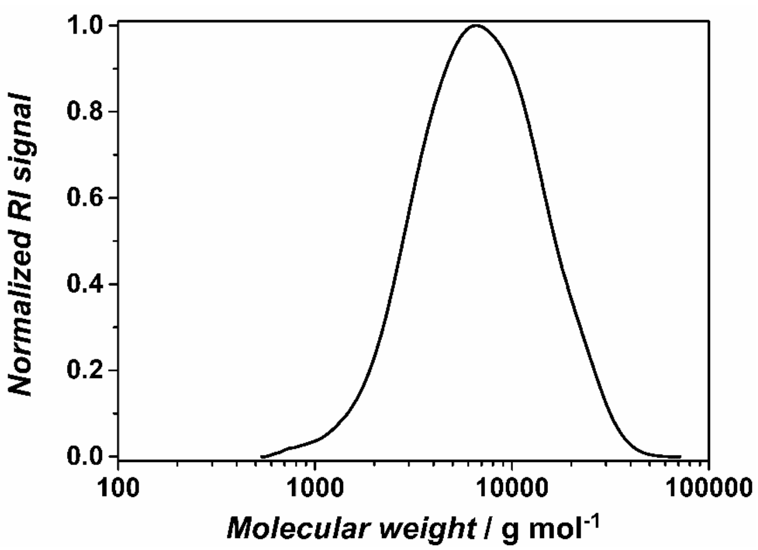

Figure S11. SEC trace of **R2** in THF using a PMMA calibration.

4.2. Photochemical Action Plot

The data for the photochemical action plots of Py-Chal and *o*MBA are taken from refs. [2c] and [6], respectively. Below is a general description of the process of recording photochemical action plots.[5] Figure S12 shows the experimental setup used to conduct the photochemical action plots. The output beam (the same light source as the printer) is initially passed through a beam expander (-50 mm and 100 mm lens combination) to ensure it is sufficiently large to uniformly irradiate the entire sample volume. The beam subsequently passes through an electronic shutter and is directed upwards using a UV silica right angle prism. Finally, the beam enters the sample vial, suspended in an aluminium block, from below. The laser energy deposited into the sample is measured above the aluminium block before and after experiments using a Coherent EnergyMax thermopile sensor (J-25MB-LE) to account for any power fluctuations during irradiation. The experiments in this study including the preparation of the solutions of Py-Chal and *o*MBA, the laser measurements and the characterization of the photochemical conversions have been described in our previous articles.[2c,6]

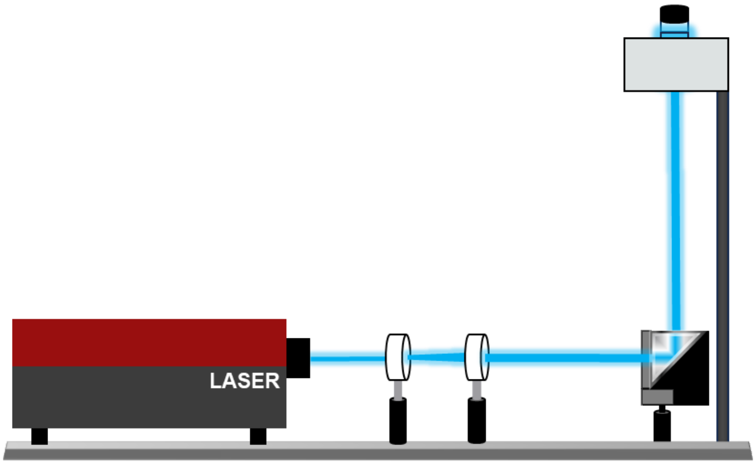

Figure S12. Schematic illustration of apparatus used for action plots and other laser experiments.

4.3. R1- and R2-based Printing

The printing methods used for **R1**- and **R2**-based printing of each of the resins on their own are the same as those for ZnTPP-based printing, utilizing however the more challenging butterfly motif (Figure S13, the G-code can be found in Supplementary files 5). The laser powers and printing speeds at different wavelengths are specified in the manuscript. After laser exposure, the objects were developed by rinsing over the glass substrate with acetone.

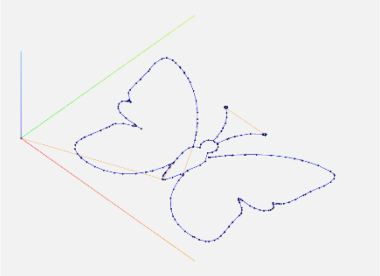

Figure S13. Images illustrating the trajectory for the butterfly generated from the G-code.

Nanoindentation tests were conducted on the butterfly structures **S1** (Py-Chal-based) and **S2** (*o*MBA-based), with measurements taken at 16 different points on each sample.

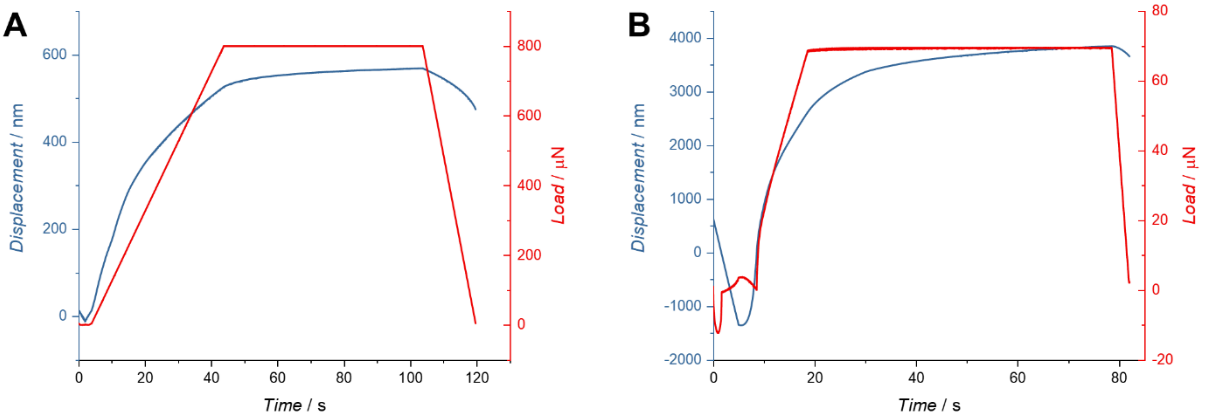

Figure S14. Displacement and load vs time curves for **S1** (B) and **S2** (A).

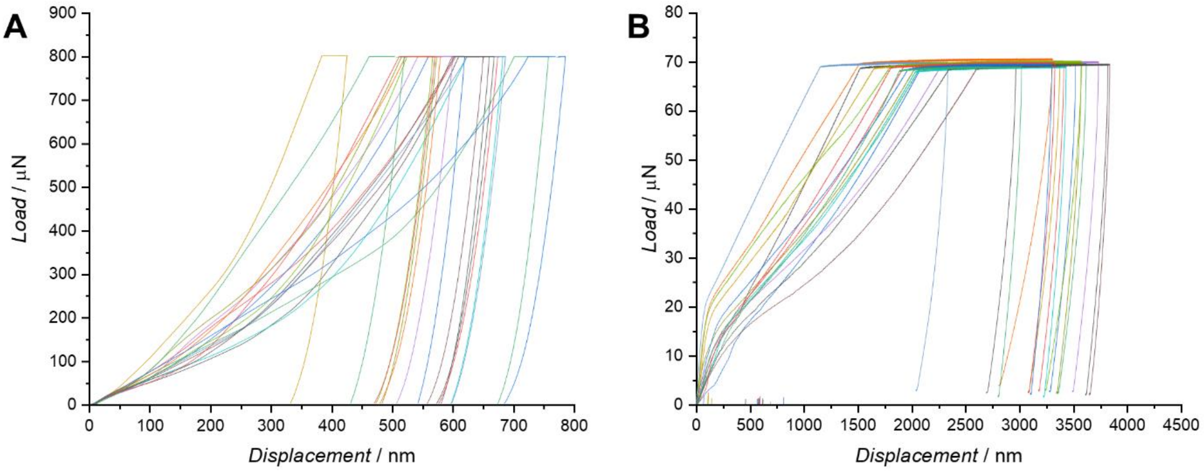

Figure S15. Indentation curves for **S1 (B)** and **S2 (A)**.

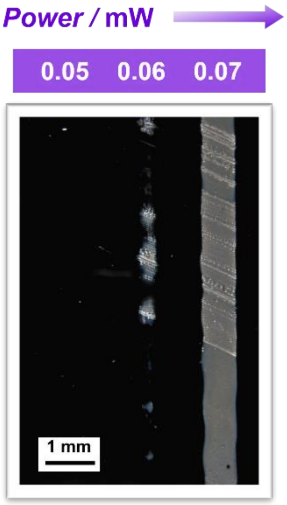

Figure S16. Microscopic image of lines printed from **R2**-based photoresist with increasing laser power *P* at 350 nm at a concentration of 70 wt% (printing speed 0.03 mm s^{-1}).

4.4. Concentration-dependence of the R1-based Photoresist on Printing Behavior

R1-based photoresists were prepared by dissolving **R1** in propylene carbonate. The dilution of the Py-Chal-based prepolymer in propylene carbonate thereby determined the response to 350 nm laser light exposure (Figure S17), revealing minimal reactivity at 60/40 (w/w) dilution and complete non-reactivity at 30/70 (w/w) dilution of **R1**/propylene carbonate. In contrast, both photoresists exhibit excellent printability at 440 nm.

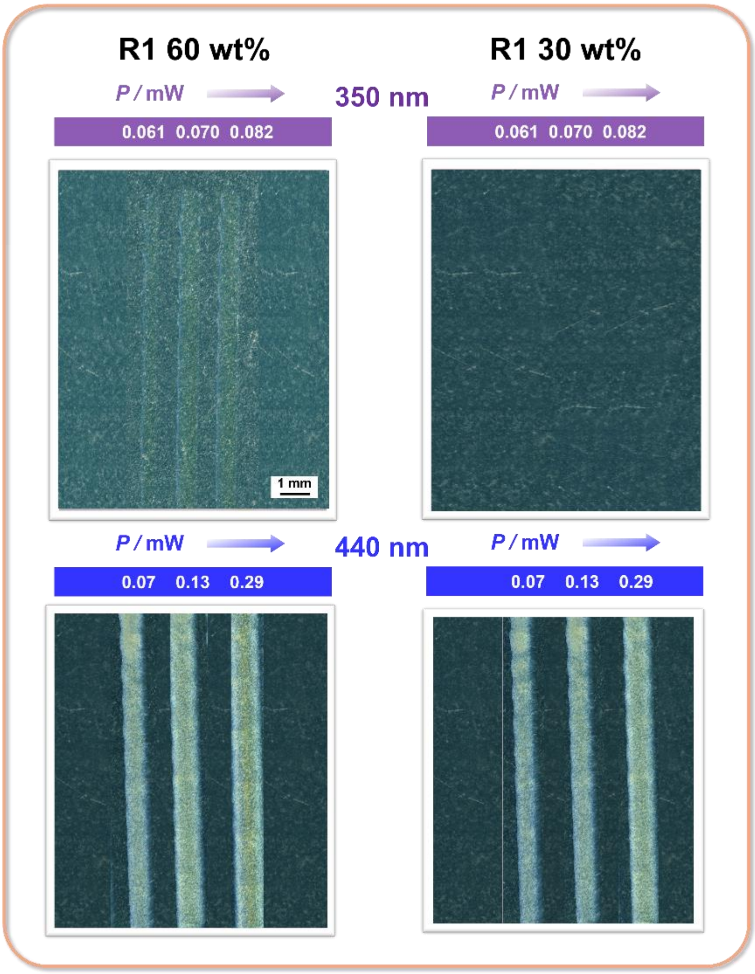

Figure S17. Optical microscopy images of lines printed from two concentrations of **R1** in **R1**-based photoresist with increasing laser power at 350 nm and 440 nm (printing speed 0.03 mm s^{-1}).

4.5. Selectivity Windows

To determine the printing conditions under which full orthogonality between **R1** and **R2** can be reached, lines were printed at three wavelengths (350, 320, 440 nm) with varying laser powers (Figure S18).

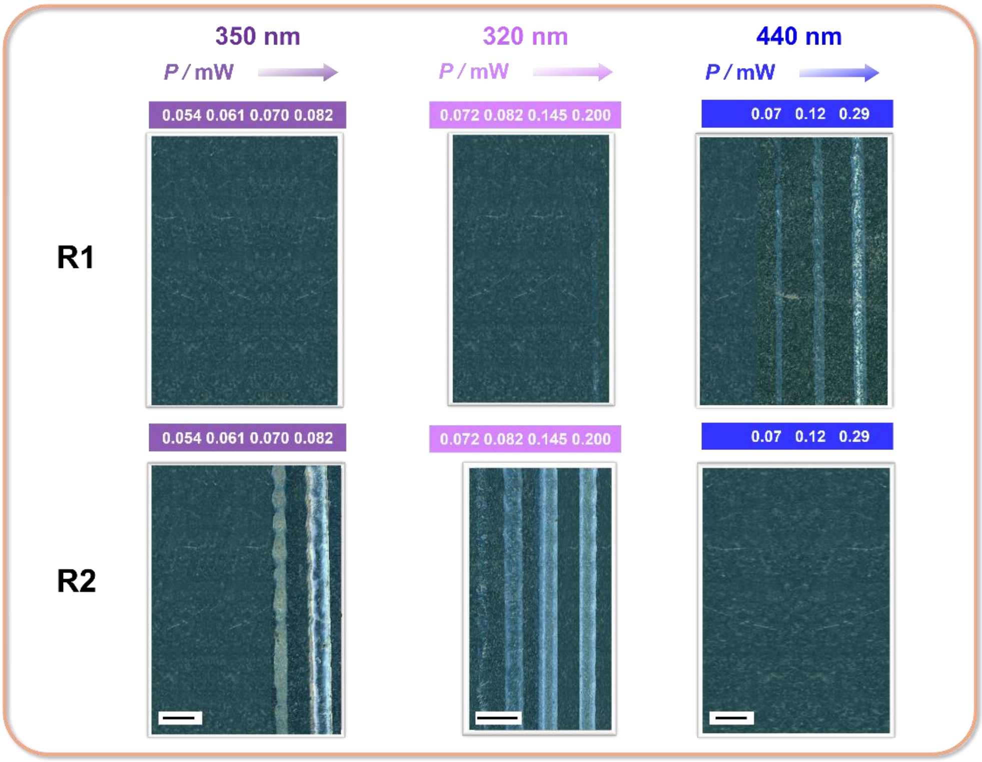

Figure S18. Optical microscopy images of lines printed from **R1**- and **R2**-based photoresists across specific power ranges at 350 nm, 320 nm, and 440 nm to determine the selectivity window.

Table S2. The beam sizes and intensities at the printing site correlated with the output power.

Wavelength / nm	Power at beam output / mW	Beam size at printing site / mm^2	Intensity at printing site / $mW\ cm^{-2}$
**320**	0.072	0.679	1.80
	0.082		2.05
	0.145		3.63
	0.2		5.01
**350**	0.054	0.887	2.98
	0.061		3.37
	0.07		3.87
	0.082		4.53
	0.09		4.97
**440**	0.07	0.997	3.16
	0.11		4.96
	0.13		5.87
	0.2		9.03
	0.29		13.09
	0.32		14.44
	0.51		23.02
	0.7		31.59
	0.81		36.56

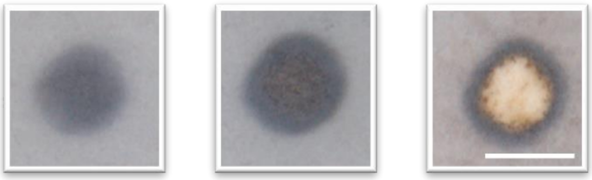

Figure S19. Spatial mapping of the laser spot at 320, 350 and 440 nm from left to right (the size of the scale bar is 1 mm).

4.6. Irradiation of Py-Chal and *o*MBA Model Compound Mixture at 440 nm and 320 nm

To confirm that full orthogonality is achieved during dual-resist-based printing, irradiation experiments with the model compounds *o*MBA and Py-Chal chromophores were conducted to analyze the reactivity via LC-MS (Figure S20). Py-Chal and *o*MBA were dissolved in ACN at a concentration of 2.8 mM and 7.4 mM, respectively (the ratio is based on the molar ratio of Py-Chal and *o*MBA units in the dual resist). The solution (300 µL in the sample vial) was placed in the previously described setup for action plots (Figure S12) and irradiated with the same number of photons (40 µmol) at 320 nm and 440 nm.

The ratio of Py-Chal dimer generated at 440 nm and 320 nm was calculated from LC traces, with a detector wavelength of 254 nm.

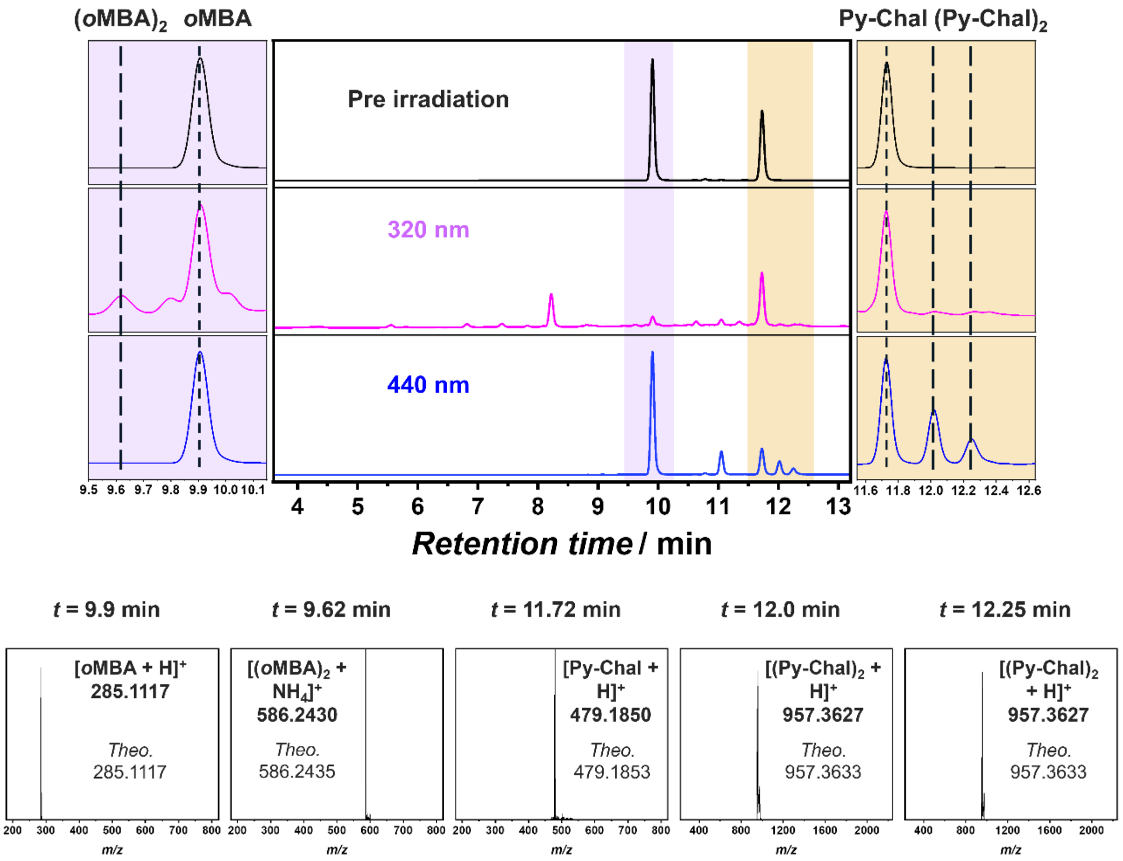

Figure S20. Comparison of the LC traces (detector wavelength 254 nm) for the Py-Chal and *o*MBA mixture before and after irradiation with 320 nm and 440 nm. Mass spectra extracted from LC-MS. Retention times are 9.9 min, 9.62 min, 11.72 min, 12.0 min and 12.25 min, corresponding to *o*MBA, (*o*MBA)$_2$, Py-Chal, (Py-Chal)$_2$ and (Py-Chal)$_2$, respectively.

4.7. Lines Printed from Dual-Resist System with Low Concentration of R1

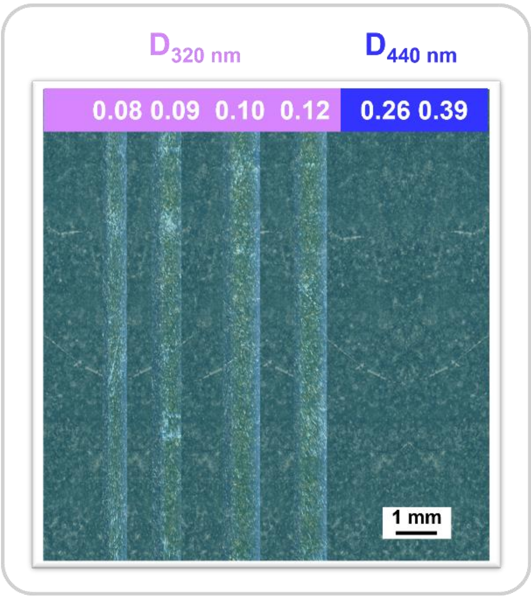

Figure S21. Optical microscopy image of lines printed from a dual-resist system with 13 wt% of **R1** (instead of 25 wt%) at 320 nm and 440 nm.

4.8. R1-based Printing with Different Solvents

Table S3. Printability of **R1**-based photoresist with different solvents at 440 nm, highlighting fabrication windows.

R1 in PC				R1 in PC + ACP			
P / mW				*P* / mW			
< 0.07	0.07	0.13	0.29	< 0.07	0.07	0.13	0.29
✗[a]	✓[b]	✓	✓	✗	✓	✓	✓

[a] lines cannot be printed; [b] lines can be printed.

4.9. Chemical Composition Mapping with FTIR

The intensity ratios of IR signals at 1150 and 1722 cm^{-1}, which correspond to the highest peaks in the spectra of **D**$_{440\ nm}$ and **D**$_{320\ nm}$, respectively, have been calculated as a function of spatial resolution across several printed lines (from left to right: first three printed at 440 nm, last two printed at 320 nm, Figure S22).

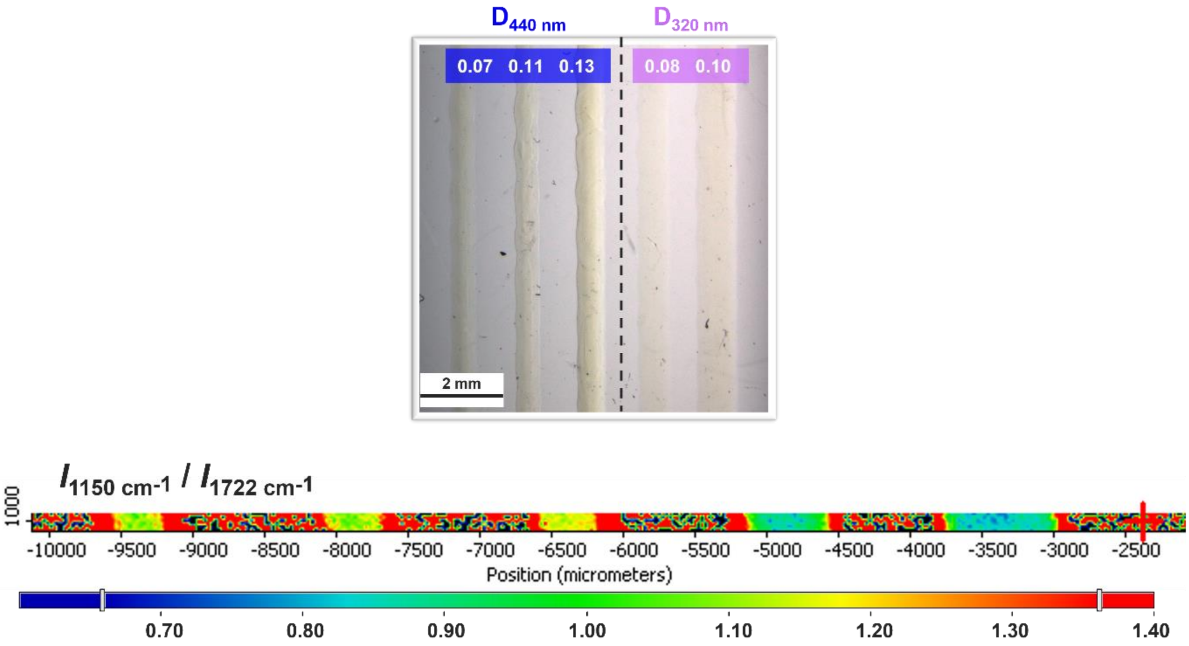

Figure S22. FTIR chemical mapping of the IR signal ratio at the wavenumbers 1150 and 1722 cm^{-1} to visualize the distinct materials printed at two different wavelengths.

4.10. Degradation of Multi-Material Structures

The samples were immersed in DMSO and placed on a stirring plate inside a Luzchem LZC-4V photoreactor. Irradiation was carried out at room temperature, using eight LZC-UVB lamps (λ_{max} = 313 nm, Figure S23) for the durations specified in the main paper.

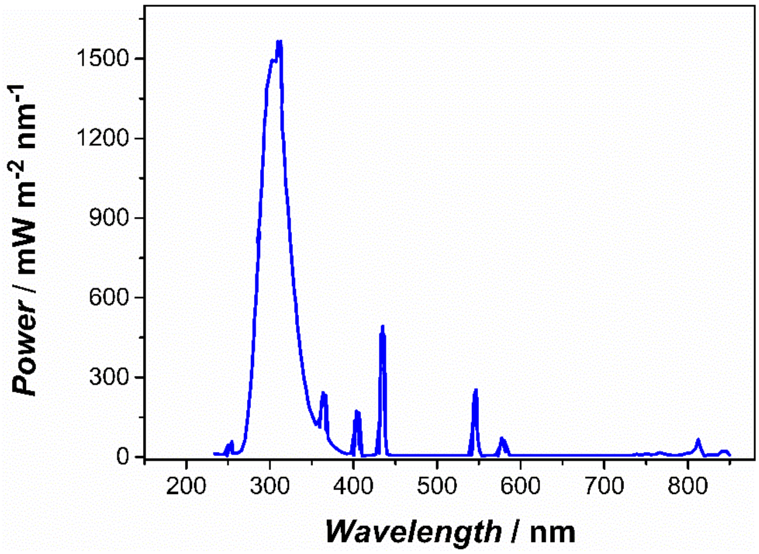

Figure S23. Emission profile of the LZC UV-B lamps used for degradation experiments.

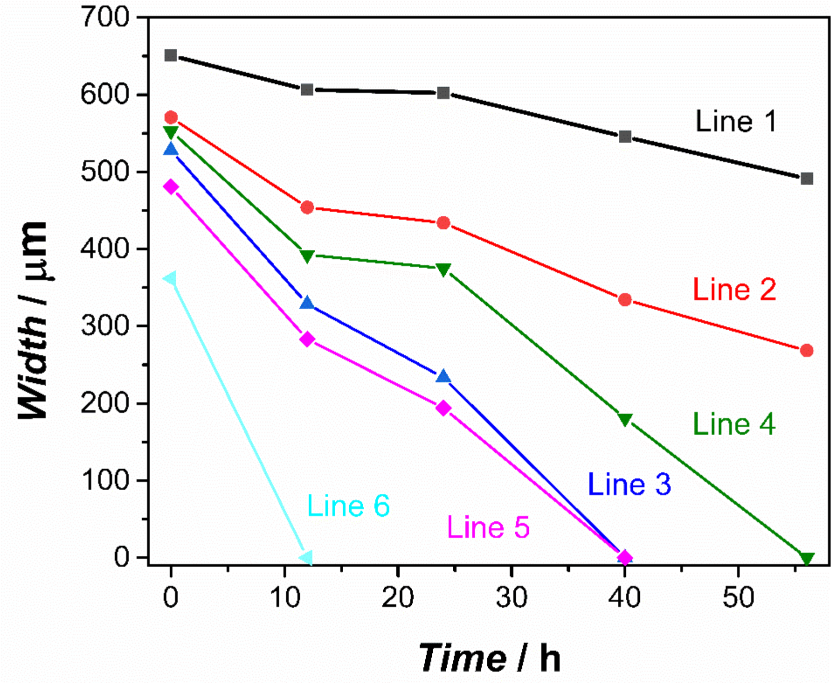

Figure S24. Widths of lines 1-6 from Figure 5A over degradation time.

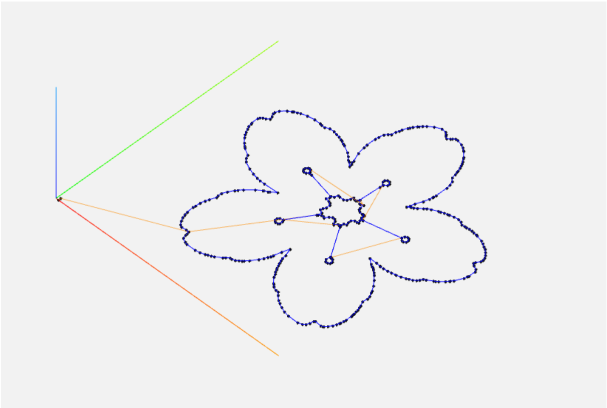

Figure S25. Image illustrating the trajectory for the cherry blossom from the G-code.

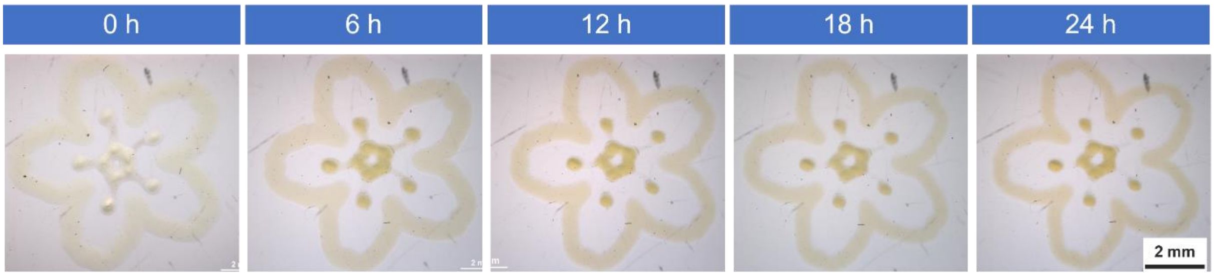

Figure S26. Cherry-blossom-like multi-material structure printed at 320 nm and 440 nm in a single print, along with its degradation over time. The center was printed at 440 nm with P = 0.072 mW (printing speed 0.09 mm s^{-1}), while the petals were printed at 320 nm with P = 0.10 mW (printing speed 0.03 mm s^{-1}). The sample was immersed in DMSO and irradiated using

UVB lamps (emission profile in Figure S23). After 12 hours, the filament almost disappeared, whereas the petal printed at 320 nm remained intact. After 24 hours, the pistil and anther degraded significantly, however, these segments were more resistant than the filament to degradation due to their increased exposure from strongly overlapping pathways (Figure S25).

References

[1] N. Awwad, A. T. Bui, E. O. Danilov, F. N. Castellano, *Chem*, **2020**, *6*, 3071.

[2] a) J. A. Carroll, F. Pashley-Johnson, H. Frisch, C. Barner-Kowollik, *Chem. Eur. J.*, **2024**, *30*, e202304174; b) F. Pashley-Johnson, R. Munaweera, S. I. Hossain, S. C. Gauci, L. Delafresnaye, H. Frisch, M. L. O'Mara, F. E. Du Prez, C. Barner-Kowollik, *Nat. Commun.*, **2024**, *15*, 6033; c) I. M. Irshadeen, K. De Bruycker, A. S. Micallef, S. L. Walden, H. Frisch, C. Barner-Kowollik, *Polym. Chem.*, **2021**, *12*, 4903.

[3] I. Mohamed Irshadeen, V. X. Truong, H. Frisch, C. Barner-Kowollik, *Chem. Comm.*, **2023**, *59*, 11959.

[4] a) A. Senthilmurugan, I. S. Aidhen, *Eur. J. Org. Chem.*, **2010**, *3*, 555; b) T. Pauloehrl, G. Delaittre, V. Winkler, A. Welle, M. Bruns, H. G. Börner, A. M. Greiner, M. Bastmeyer, C. Barner-Kowollik, *Angew. Chem., Int. Ed.*, **2012**, *51*, 1071; c) T. K. Claus, B. Richter, V. Hahn, A. Welle, S. Kayser, M. Wegener, M. Bastmeyer, G. Delaittre, C. Barner-Kowollik, *Angew. Chem., Int. Ed.*, **2016**, *55*, 3817; d) S. Bialas, L. Michalek, D. E. Marschner, T. Krappitz, M. Wegener, J. Blinco, E. Blasco, H. Frisch, C. Barner-Kowollik, *Adv. Mater.*, **2019**, *31*, 1807288.

[5] a) I. M. Irshadeen, S. L. Walden, M. Wegener, V. X. Truong, H. Frisch, J. P. Blinco, C. Barner-Kowollik, *J. Am. Chem. Soc.*, **2021**, *143*, 21113; b) S. L. Walden, J. A. Carroll, A.-N. Unterreiner, C. Barner-Kowollik, *Adv. Sci.*, **2024**, *11*, 2306014.

[6] J. P. Menzel, B. B. Noble, A. Lauer, M. L. Coote, J. P. Blinco, C. Barner-Kowollik, *J. Am. Chem. Soc.*, **2017**, *139*, 15812.
